# Design of a Multi-Epitope Vaccine Candidate Against Infectious Laryngotracheitis Virus Affecting Poultry by Computational Approaches

**DOI:** 10.3390/biology14070765

**Published:** 2025-06-25

**Authors:** Periyasamy Ponnusamy, Kuppannan Sukumar, Angamuthu Raja, Sellappan Saravanan, Palani Srinivasan, Kalaivanan Ramya, Mani Selvaraju, Ramasamy Saravanan

**Affiliations:** 1 Department of Veterinary Microbiology, Veterinary College and Research Institute, Namakkal 637002, Tamil Nadu, India; 2 Department of Animal Biotechnology, Madras Veterinary College, Chennai 600007, Tamil Nadu, India; 3 Department of Veterinary Pathology, Veterinary College and Research Institute, Namakkal 637002, Tamil Nadu, India; 4 Veterinary College and Research Institute, Namakkal 637002, Tamil Nadu, India; 5 Department of Animal Genetics and Breeding, Veterinary College and Research Institute, Tamil Nadu Veterinary and Animal Sciences University, Namakkal 637002, Tamil Nadu, India

**Keywords:** infectious laryngotracheitis virus, glycoprotein B, glycoprotein D, immunoinformatics, multi-epitope vaccine, avian beta-defensin 1

## Abstract

Infectious laryngotracheitis (ILT) is a severe upper respiratory disease highly contagious in chickens that causes a huge economic impact on the poultry industry all over the world. The current study aimed to design a multi-epitope-based vaccine candidate using envelope glycoprotein B and glycoprotein D of the ILT virus using an immune informatics approach. The glycoproteins B and D are crucial for attachment as well as entry of ILT virus inside the cell, which makes them a potential option for designing vaccine candidates. The prediction of epitopes, viz. helper T lymphocyte, cytotoxic T lymphocyte and interferon-gamma producing epitopes, was performed and high-scoring predicted epitopes were joined in an organized manner using suitable linkers to design the final vaccine candidate. The avian beta-defensin 1 was included as an adjuvant in the amino-terminal of the vaccine design that possesses antimicrobial activity and histidine residues at the carboxy-terminal for the purpose of purification. The final vaccine candidate was evaluated for its physicochemical characteristics, solubility, antigenicity, stability, and allergenicity and validated for its modeling. Molecular docking, binding affinity, and interacting residues between the vaccine candidate and immune receptors, viz. TLR 3, MHC Class I and Class II were assessed. Further, to assess the immune response profile generated by the final vaccine design, an insilico immune simulation study was also performed. The findings of this study revealed that the final vaccine candidate was antigenic, nonallergenic, stable, interacted with immune receptors, and able to produce antibodies as well as cellular immune responses against ILTV infection.

## Simple Summary

The occurrence of infectious diseases has an impact on the poultry industry’s phenomenal growth rate. Infectious laryngotracheitis (ILT) is among the diseases that cause severe respiratory disease in chickens, which affects the poultry industry worldwide. The major drawbacks of conventional vaccines are inherent virulence, latency and low immunogenicity, which demands the need to generate alternative vaccines to combat ILT infection. In this study, a multi-epitope-based vaccine construct using envelope glycoproteins B and D of the ILTV was designed by computational approaches. The results of the study showed that the final vaccine candidate was antigenic, nonallergenic, stable, interacted with immune receptors, and able to produce cellular and antibody immune responses against ILTV infection, analyzed by immunoinformatic approaches. These findings offer a base for the development of alternatives to traditional vaccines to combat ILTV infection in poultry.

## 1. Introduction

Infectious laryngotracheitis is considered a highly contagious and severe acute respiratory tract infection in chickens that leads to substantial production losses due to high mortality and lowered production performances [1]. ILT is primarily an infection of chickens but also infects peafowl, pheasants, and turkeys [2,3]. The virus causing ILT has been classified under the family *Herpesviridae*, subfamily *Alphaherpesvirinae*, genus *Iltovirus* and species *Gallidherpesvirus*1 [4]. The ILT virus particles were reported to be pleomorphic with variations in size ranging from 200 to 350 nm and possess an icosahedral capsid measuring about 100 nm in diameter [5], which is enclosed by a tegument layer and outer envelope [6]. The genome was also observed to be typical of Baltimore group I—a linear double strand of DNA having 150–155 kb nucleotides. ILT virus is enveloped and remains sensitive to ether, chloroform, lipolytic solvents, and oxidizing agents [7,8,9].

The genomic DNA has also been reported to encode a unique long (UL), unique short (US), and two inverted repeats (IR) sequences [10,11]. The genome has also been reported to have 80 open reading frames (ORFs). Among these 65, 9 and 6 ORFs were located in the UL, US, and IR regions, respectively [10,12,13].

The viral envelope was reported to be comprised of glycoproteins, namely B, C, D, E, G, H, I, J, K, L, and M, which are encoded by ORFs of UL27, UL44, US6, US8, US4, UL22, US7, US5, UL53, UL1, and UL10 respectively [14]. The surface envelope glycoproteins of the ILT virus are required for entry, genome replication, and release. Further, it appears to be involved in the modulation of antibody and cell-mediated immune responses in the host [6]. The glycoprotein B is considered to be one of the major proteins of the ILT virus and plays a crucial role in the virus’s adherence and entry into the target cells [15]. At the same time, the glycoprotein D encoded by US6 is required for virus replication and plays a main role in the adherence and entry of the virus into the cells. The glycoproteins B and D have been shown to produce neutralizing antibodies and also act as targets for a recombinant vaccine against ILTV [16].

Traditional vaccines, such as chicken embryo origin (CEO) attenuated and tissue culture origin (TCO) attenuated live vaccines, are generally used in commercial poultry flocks worldwide. However, reverting to virulence, latency and increasing their virulence by bird-to-bird passage are the limitations associated with conventional live-modified vaccines against ILTV [17]. Nowadays, multi-epitope-based chimeric/subunit vaccines are reported to be more attractive vaccine candidates and have many advantages over conventional vaccinology because of their easier construction as well as production, safer due to the absence of entire pathogen, cheaper, and consume less time as they do not need culturing of pathogens, highly specific and also stable [18]. Further, conventional vaccinology needs whole organisms or large proteins, resulting in possibilities of allergic reactions along with unwanted antigenic load, which can be avoided by vaccines based on peptides that consist of short immunity-inducing epitopes. The peptide-based vaccine was demonstrated to induce strong and targeted antibody responses and cellular immune responses without allergic responses by computational biology [19]. Further, the final chimeric vaccine construct was screened for its secondary, tertiary structure analysis, molecular docking studies with immune receptors, immune simulation analysis, and physicochemical parameters like antigenicity, toxicity, solubility, and thermostability to validate the final vaccine candidate using various immunoinformatics tools. The main focus of the present study was to design a potential multi-epitope subunit/chimeric vaccine candidate using promiscuous cytotoxic and helper T cell epitopes against ILTV by computational approaches. The proposed vaccine candidate is then analyzed for its physicochemical parameters, immune response profile, binding affinity, and interactive residue studies with innate and adaptive immune receptors.

## 2. Materials and Methods

### 2.1. Retrieval of ILTV’s Glycoprotein B and D Amino Acid Sequences

The NCBI database was used to retrieve 67 and 56 amino acid sequences of glycoprotein B and glycoprotein D of ILT virus, respectively. Multiple sequence alignment was performed in the amino acid sequences retrieved from NCBI to determine the conservation of the proteins. Additionally, the consensus sequence was created for both glycoproteins B and D of ILTV using BioEdit version 7.2.5 sequence alignment editor developed by Tom hall, Roseau Valley, Dominica.

### 2.2. Physicochemical Parameters Evaluation

The consensus sequence of both glycoproteins B and D of ILTV was subjected to analysis of physicochemical properties, viz., molecular weight, theoretical pI, and half-life in mammalian reticulocytes in vitro using the ProtParam online tool (http://web.expasy.org/protparam, accessed on 7 April 2020) [20].

### 2.3. Homology Modeling

The consensus sequence of both glycoproteins B and D of ILTV was subjected to modeling of three-dimensional (3D) protein structures using the RaptorX structure prediction server (http://raptorx.uchicgo.edu/StructurePrediction/predict/, accessed on 6 May 2020) [21]. The 3D models of both glycoproteins B and D of ILTV were further refined by using a galaxyrefine server (http://galaxy.seoklab.org/cgi-bin/submit.cgi?type=REFINE, accessed on 20 July 2020) [22] and analyzed the quality and reliability of the model using the RAMPAGE server (http://mordred.bioc.cam.ac.uk/~rapper/rampage.php, accessed on 20 July 2020) [23].

### 2.4. Cytotoxic T-Cell Lymphocytes (CTL) Epitopes Prediction

The 9-mer length CTL epitopes were predicted by using the NetCTL.1.2 online server on the basis of an artificial neural network algorithm (https://www.cbs.dtu.dk/services/NetCTL, accessed on 9 April 2020). The CTL epitopes were predicted based on the combined score of MHC binding affinity, proteasomal C-terminal cleavage, and Transporter Associated with Antigen Processing (TAP) transport efficiency. The highest-scoring CTL epitopes recognized by HLA Class I supertypes in humans were selected. The thresholds for parameters like epitope identification, proteasomal C-terminal cleavage, and TAP were set at 0.75, 0.15, and 0.05, respectively, as described previously [24]. Chicken BF1 and BF2 regions are referred to as class I MHC molecules since chicken MHC alleles are not available in the immunoinformatic databases. However, chicken MHC and HLA alleles were found to be similar in the presentation of antigens as well as immune response induction.

### 2.5. Antigenicity, Allergenicity, Toxicity Analysis and Conservancy Prediction of CTL Epitopes

The predicted CTL epitopes of glycoproteins B and D of ILTV were examined for antigenic potential using the VaxiJen v2.0 online tool (https://www.ddg-pharmfac.net/VaxiJen/VaxiJen.html, accessed on 19 July 2020). The threshold value for antigenicity prediction was set to 0.4 [25]. The peptides having a VaxiJen score of more than 0.4 were selected and further categorized for their immunogenic potential using the Immune Epitope Database (IEDB) tool (https://www.iedb.org, accessed on 19 July 2020) [26]. The peptides having a VaxiJen score of less than 0.4 were discarded. The allergenic and nonallergenic potential of the predicted CTL epitopes of glycoproteins B and D of ILTV was evaluated using the AllerTop v2.0 tool (https://www.ddg-pharmfac.net/AllerTOP, accessed on 19 July 2020) [27]. The toxicity of the predicted CTL epitopes of these proteins of ILTV was analyzed using the server ToxinPred (https://crdd.osdd.net/raghava/toxinpred, accessed on 19 July 2020). According to all physicochemical parameters, this website provides confirmation that epitopes are non-toxic to the host. The threshold value was set as the default for analyzing the toxicity of the peptides using the ToxinPred server. The conservation of the predicted CTL epitopes of these proteins of ILTV was evaluated by using the Immune Epitope Database (IEDB).

### 2.6. Helper T Cell (HTL) Epitopes Prediction

The 15-mer length HTL epitopes were predicted for glycoproteins B and D of ILTV using the Net MHC II pan 3.2 server (www.cbs.dtu.dk/services/NetMHCIIpan, accessed on 9 April 2020). The HTL epitopes recognized by HLA Class II DRB1 alleles, such as 01:01, 03:01, 04:01, 07:01, 08:03, 10:01, 11:01, 12:01, 13:02, 14:01 and 15:01, were selected. The HTL epitopes were sorted into strong binders, intermediate binders, and non-binders based on the idea of percentile rank. The threshold value was set at 2%, 10%, and greater than 10% for strong, weak, and non-binding epitopes, respectively [28].

### 2.7. Antigenicity, Allergenicity and Toxicity Analysis of HTL Epitopes

The predicted HTL epitopes of glycoproteins B and D of ILTV were analyzed for antigenicity, allergenicity and toxicity analysis using the VaxiJen v2.0 online tool [25], AllerTop v2.0 tool [29] and ToxinPred server, respectively, similar to that of CTL epitopes. Toxicity analysis was performed to find out if any predicted CTL, as well as HTL epitopes, are toxic or non-toxic. In the present study, all predicted epitopes were subjected to toxicity analysis, and only non-toxic epitopes were selected for the final vaccine construct against ILTV. The ToxinPred server was widely used in-silico studies to predict the toxicity of peptides or proteins, and is also able to spot the toxic regions in proteins as well as designthe least toxic peptides by machine learning approaches, motif analysis and similarity-based approaches.

### 2.8. Interferon (IFN-γ) Epitopes Prediction

The IFNepitope server (https://crdd.osdd.net/raghava/ifnepitope, accessed on 19 July 2020) was used to determine IFN-γ-inducing epitopes for both glycoproteins B and D of ILTV. These epitopes are vital for immunological modulation as well as antiviraland antitumor processes. To determine the presence of the IFN gamma epitope, all HTL epitopes were utilized as inputs to identify the IFN gamma epitopes. Methods such as motive-based, accuracy hybrid analysis and machine learning strategy were used to predict the IFN-γ epitopes [30].

### 2.9. Linear B Cell Epitopes Prediction

Using IEDB, the linear/continuous B cell epitopes were determined. Additionally, the surface accessibility and antigenicity of the predicted B cell epitopes were also evaluated. IEDB does not contain chicken data sets. However, the IEDB databases are already used to predict T cell epitopes using human reference allele sets for poultry pathogens like Newcastle disease virus, Hydropericardium syndrome, Chicken infectious anemia, *Mycoplasma gallisepticum* and Avian leukosis virus since the human allele sets were reported to be similar in functional actions like antigen presentation and stimulation of immune response. This database has also been used to predict B cell epitopes for different poultry pathogens such as Newcastle disease virus, infectious bronchitis virus (IBV) and influenza virus.

### 2.10. Mapping of CTL and HTL Epitopes

The predicted CTL and HTL epitopes of both glycoproteins B and D were mapped into respective 3D protein models using the UCSF Chimera visualization tool.

### 2.11. Designing of Multi-Epitope Final Vaccine Construct

The final multi-epitope vaccine was constructed by joining the predicted peptide sequences of both glycoproteins B and D using suitable linkers in an organized manner. The AAY linker was utilized to join together the predicted CTL epitopes of glycoproteins B and D. The glycine proline-rich linkers or GPGPG were employed to join the HTL epitopes of glycoproteins B and D. As an adjuvant, the Avian beta-defensin sequence was included into the amino terminal of the vaccine candidate linked by the EAAAK linker. Further, His-tag was used in the final vaccine construct at the carboxy-terminal for purification purposes [31].

### 2.12. Physiochemical Parameters Evaluation of the Final Vaccine Design

The ProtParam online tool was utilized to evaluate the physicochemical properties of the final vaccine design, viz., molecular weight, theoretical pI, instability index, aliphatic index, GRAVY (Grand average of hydropathicity), and half-life. The solubility, antigenicity, and allergenicity of the final vaccine design were assessed by servers, viz. SOLpro [32], VaxiJen v2.0, and AllerTOP, respectively.

### 2.13. Homology Modeling of the Final Vaccine Design

Online tools such as PSIPRED and PDBsum were employed to predict the secondary structure of the final vaccine design. The 3D modeling for the final vaccine candidate was performed by using the 3Dpro server [32]. The galaxyrefine server was used to further refine the 3D model of the final vaccine design. Then, the refined 3D model was subjected to validation using the RAMPAGE server for the quality and reliability of the model and ProSA [33]. The finalized vaccine candidate model was further validated using ERRAT scores for verification [34].

### 2.14. Molecular Docking

Using the Cluspro server, the final vaccine design was put through a molecular docking analysis with the Toll-like receptor 3 (TLR 3). Molecular docking was performed to analyze the interaction of multi-epitope vaccine candidates with innate immune receptors such as TLRs to elicit an immunological response. The TLR 3 structure (PDB ID:2a0z) was recovered from the Protein Data Bank. In addition, the multi-epitope vaccine design was docked with chicken adaptive immune receptors such as MHC class I and class II. The class I and class II amino acid sequences of chicken MHC receptors were obtained from NCBI. The 3D models for both class I and class II MHC antigens were created using a 3D pro server and further refined by using a galaxyrefine server.

### 2.15. Binding Affinity Analysis

The final vaccine candidate’s binding affinity analysis with TLR 3, class I and class II MHC receptors was performed by the PRODIGY webserver [35,36]. Then, mapping of interacting residues of the final vaccine construct with TLR 3, class I and class II MHC receptors was conducted using PDBsum (https://www.ebi.ac.uk/thornton-srv/databases/pdbsum/Generate.html, accessed on 18 August 2020) [37].

### 2.16. Immune Simulation

Using the C-ImmSimserver (http://150.146.2.1/C-IMMSIM/index.php, accessed on 23 August 2020), the immunological simulation of the final vaccine candidate was conducted to assess its immunogenicity and antibody and cell-mediated immune profile. Single dose as well as three doses of prophylactic vaccine were given at 10 days intervals. The entire simulation was set at 1000 steps [38].

### 2.17. Codon Optimization

The codon optimization and reverse translation of the final vaccine design were performed using JCat, a Java codon optimization tool (https://www.jcat.de, accessed on 21 August 2020). The DNA sequence of the final vaccine design was obtained, and the *E. coli* K12 strain was chosen as the source organism for efficient expression [39].

## 3. Results

### 3.1. Retrieval of Glycoproteins B and D Sequences

ILTV’s glycoproteins B and D were selected in the present study to design a multi-epitope vaccine construct against ILT infection. The amino acid sequences of both proteins were obtained from the NCBI database. Multiple sequence alignment of glycoprotein B had seven amino acid variations at residues R44H, A116V, M348T, M551V, I644T, P799S, and K805R. The glycoprotein D had ten amino acid variations at residue positions P4S, K21E, V194L, P198S, V202L, T215N, Q251R, R291G, D368N, and M407I during multiple sequence alignment by BioEdit software. The majority of residues present at variation sites were taken into consideration for the creation of a common sequence against each protein. Glycoprotein B and glycoprotein D had sequence lengths of 883 and 434 amino acids, respectively. The physicochemical properties were evaluated for both the proteins shown in Table 1.

### 3.2. CTL Epitope Prediction

**Figure 1:**
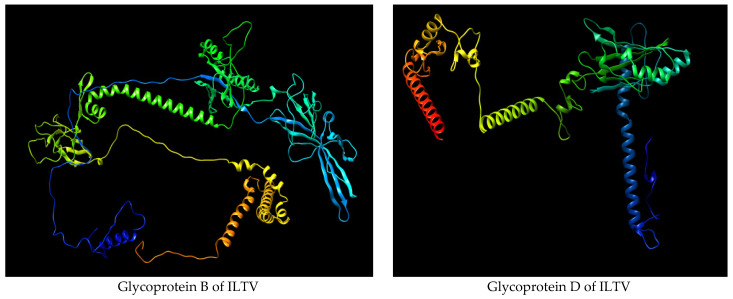
Tertiary structure of glycoprotein B and glycoprotein D of ILTV.

**Figure 2:**
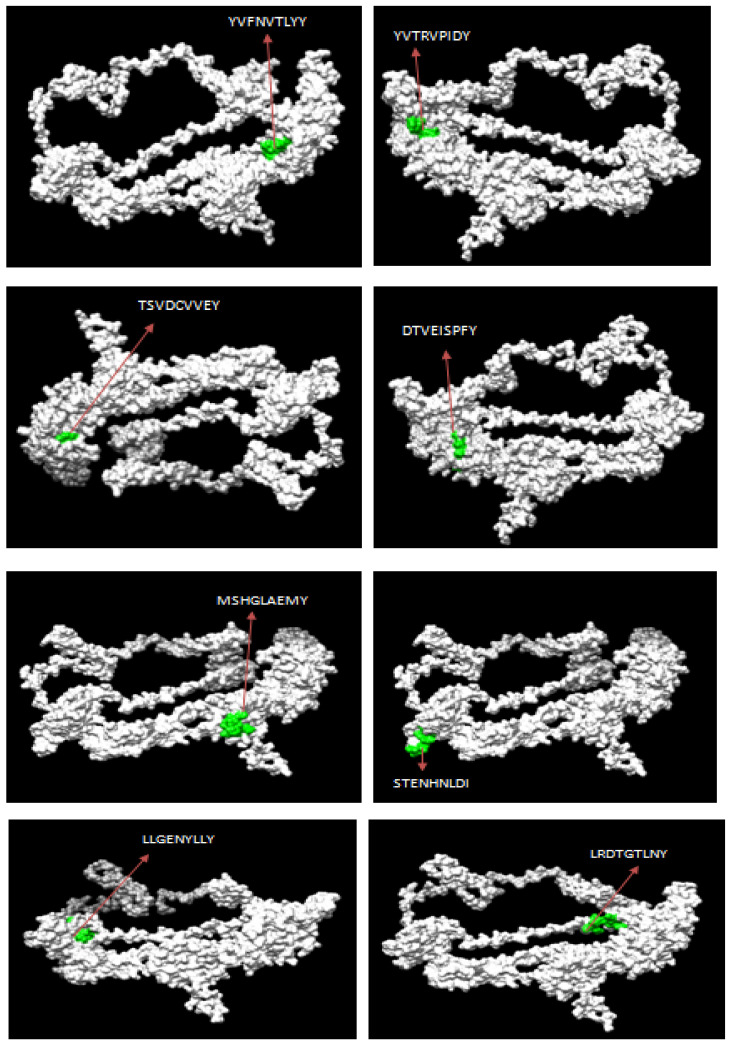
Mapping of predicted CTL epitopes in the 3D structure of glycoprotein B of ILTV.

**Figure 3:**
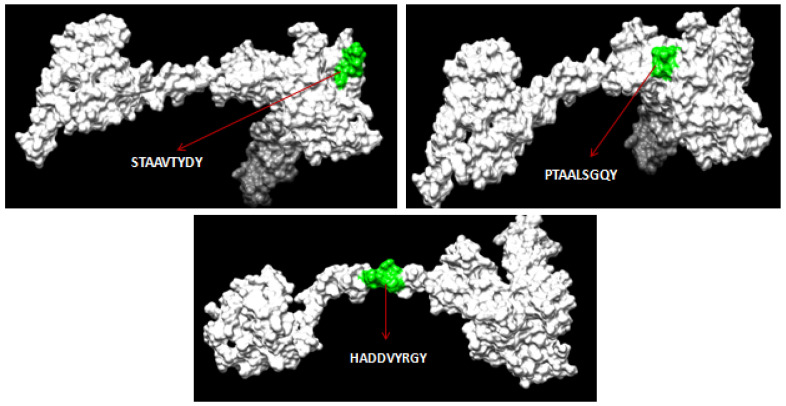
Mapping of predicted CTL epitopes in the 3D structure of glycoprotein D of ILTV.

The Net CTL1.2 server was used to predict a total of 27 and 10 high-scoring CTL epitopes for ILTV’s glycoproteins B and D, respectively. The best CTL epitopes were filtered based on the combined score of MHC binding affinity, proteasomal C-terminal cleavage, and TAP with default set values. Further, the epitopes were screened out for antigenicity, allergenicity, toxicity, and conservancy using VaxiJen v2.0, AllerTOP, Toxinpred, and IEDB, respectively. The three-dimensional (3D) structures for both glycoproteins B and D were modeled by the RaptorX server and further refined using the Galaxy Refine server (Figure 1). The filtered CTL epitopes for the vaccine design were mapped with the 3D structure of the respective proteins (Figure 2 and Figure 3).

### 3.3. HTL Epitope Prediction

**Figure 4:**
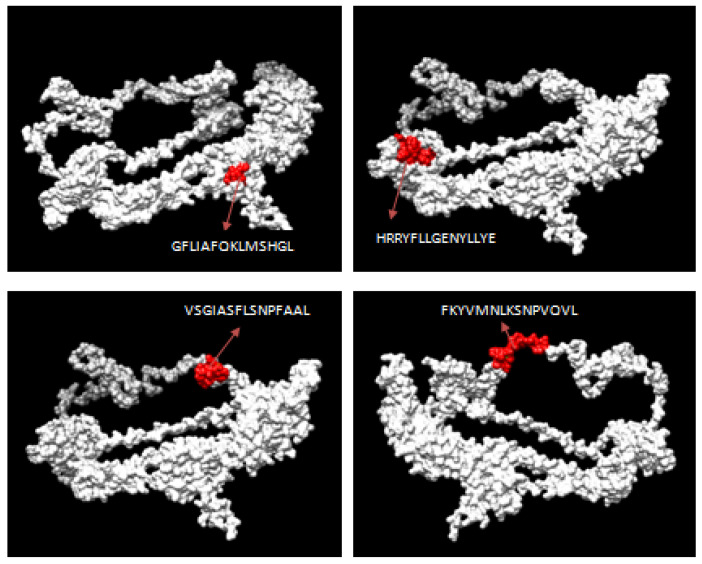
Mapping of predicted HTL epitopes in the 3D structure of glycoprotein B of ILTV.

**Figure 5:**
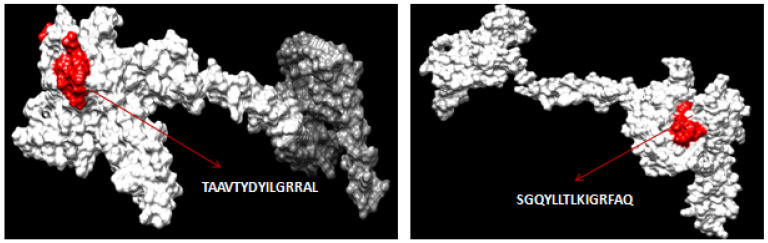
Mapping of predicted HTL epitopes in the 3D structure of glycoprotein D of ILTV.

Using NetMHC II pan 3.2 servers, 81 and 11 numbers of 15-mer length HTL epitopes were first predicted based on the glycoproteins B and D’s strong binding affinity to class II MHC alleles, respectively. Among the predicted epitopes, the best HTL epitopes were filtered based on binding with several HLA DRB1 alleles with strong affinity and antigenicity. The antigenic, nonallergenic, non-toxic and highly conserved HTL epitopes were chosen for the final vaccine construct. The three-dimensional (3D) structures for both glycoproteins B and D were modeled by the RaptorX server and further refined using the Galaxy Refine server. The selected HTL epitopes for the vaccine construct were mapped with the 3D structure of the respective proteins (Figure 4 and Figure 5).

### 3.4. IFN-γ Inducing Epitopes Prediction

The HTL epitopes of both glycoproteins were subjected to IFN gamma-inducing epitope prediction using the IFNepitope server. The interferon-gamma-inducing epitopes were shown in the Supplementary files for both glycoproteins B and D of ILTV.

### 3.5. Designing the Final Multi-Epitope Vaccine Construct

**Figure 6:**
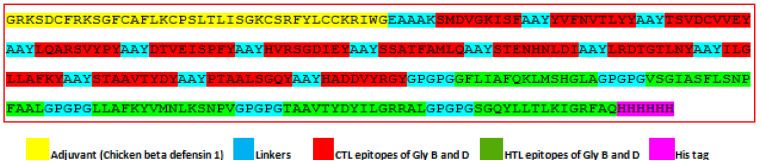
Design of multi-epitope final vaccine construct against ILTV.

The final multi-epitope vaccine contains four domains, including an adjuvant, CTL, HTL, and His tag. The final multi-epitope vaccine is composed of 304 amino acids. At the vaccine construct’s N terminus, the adjuvant chicken beta-defensin 1 with an amino acid sequence of GRKSDCFRKSGFCAFLKCPSLTLISGKCSRFYLCCKRIWG was added using the helical EAAAK linker. The final vaccine construct contains thirteen CTL epitopes of glycoproteins B and D of ILTV, which were added next to the chicken beta-defensin 1 adjuvant. Then, five HTL epitopes, including interferon-gamma-inducing epitopes, were added next to CTL epitopes. The tag consists of six histidine residues (HHHHHH), which were added at the C-terminal of the final vaccine construct for purification. The adjuvant, CTL, and HTL epitopes were joined by suitable linkers such as the EAAAK linker between adjuvant and CTL epitopes, the AAY linker between CTL epitopes, and the GPGPG linker between HTL epitopes, as shown in Figure 6.

### 3.6. Evaluation of Physicochemical Properties of Final Vaccine Design

The final vaccine construct was evaluated for its physicochemical properties using the ProtParam tool provided by the Expasy server. The molecular weight was computed to be 32.76 kDa, which shows the ideal range for a prophylactic vaccine candidate. The physicochemical properties of the final vaccine construct are shown in Table 2.

### 3.7. Secondary and Tertiary Structure Modeling

**Figure 7:**
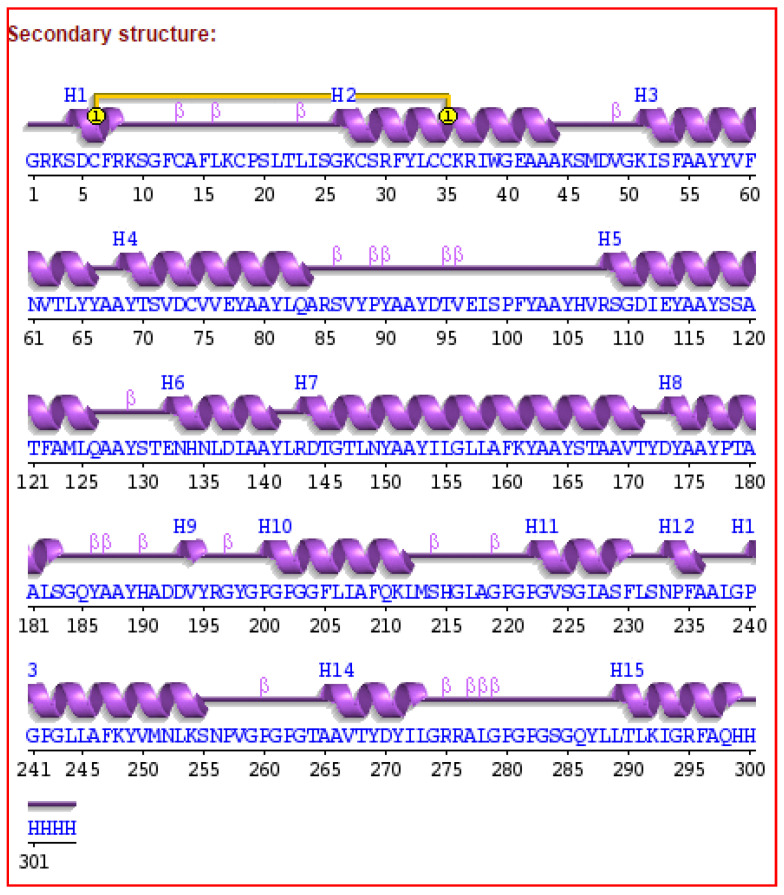
Secondary structure of the multi-epitope final vaccine construct against ILTV.

**Figure 8:**
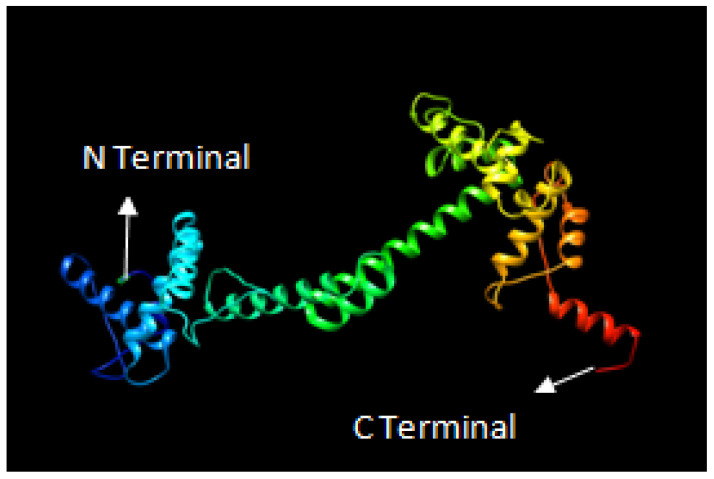
A 3D model of the multi-epitope final vaccine construct against ILTV.

**Figure 9:**
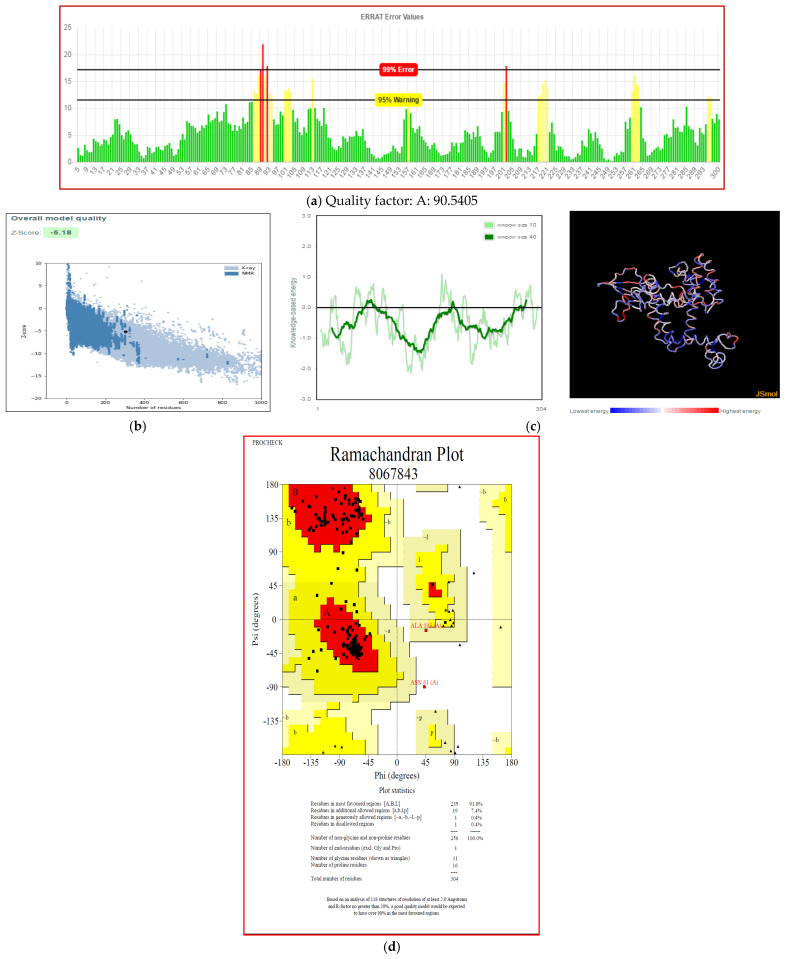
Validation of a 3D model of multi-epitope final vaccine construct (**a**) Validation of structure by ERRAT (**b**) Validation of structure by ProSA (**c**) Validation of structure by ProSA—Local model quality (**d**) Validation of a 3D model of final vaccine construct by Ramachandran Plot analysis.

The secondary structure of the final vaccine construct was determined using PDBsum. The final vaccine construct had 15 helices, 21 beta turns, 17 helix-helix interactions, and one disulfide bond (Figure 7). Furthermore, the 3D model for our vaccine design was generated using the 3Dpro server. The 3Dpro server predicted the tertiary structure based on the de novo method to make 3D models and was further refined by using the galaxyrefine server. The best refined 3D model is shown in Figure 8. The Ramachandran plot analysis was used to validate the refined 3D model, which showed 91.8%, 7.8%, and 0.4% residues in the favored region, allowed region, and disallowed region, respectively. The refined 3D model of our vaccine construct had an ERRAT score of 90.5405. Further, the overall quality of the 3D model was assessed by Z score using PROSA, which showed a Z score of −5.18 for our final vaccine construct (Figure 9a–d).

### 3.8. Molecular Docking with Immune Receptors

**Figure 10:**
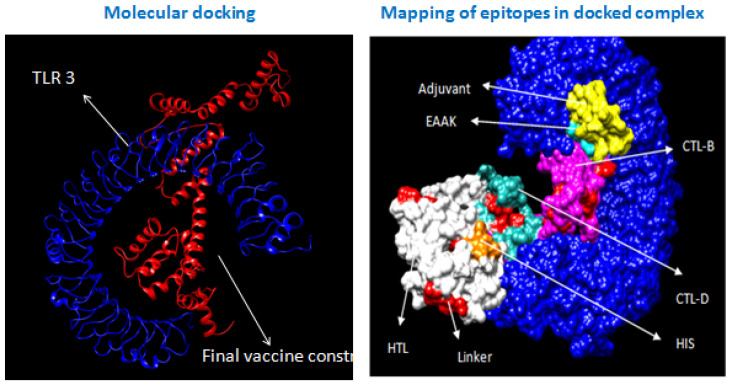
Molecular docking of the final multi-epitope vaccine with TLR3.

**Figure 11:**
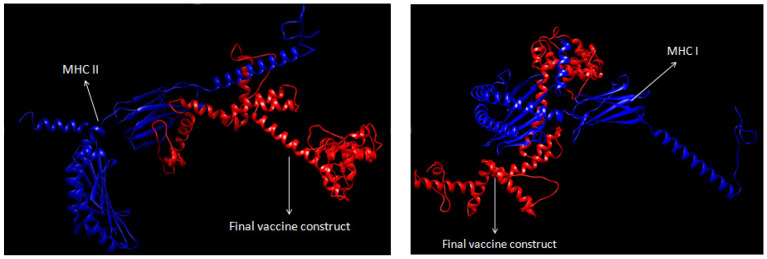
Molecular docking of the final multi-epitope vaccine with Class II MHC and Class I MHC.

Molecular docking of TLR3 with the final vaccine construct using a cluspro server produced the top 10 protein-protein docking structures with binding energy (Figure 10). The TLR 9 primarily recognizes DNA viruses containing CpG motifs in mammals. Whereas chickens lack a gene encoding TLR 9 and TLR 21, which is equivalent to TLR 9 present in chickens, the TLR 21 structure is not available in the protein data bank. Therefore, TLR 3 was selected for molecular docking analysis with our final vaccine construct. The best-docked protein-protein complex was chosen based on the score of negative energy. In addition, molecular docking analysis of the final vaccine construct with class I and II MHC receptors using a cluspro server was carried out to study the interaction of epitopes, including CTL and HTL, with respective Class I and Class II MHC, resulting in the generation of cell-mediated as well as humoral immune responses respectively (Figure 11).

### 3.9. Binding Affinity Analysis

The binding affinity of the docked complexes, such as vaccine + TLR3, vaccine + class I MHC, and vaccine + class II MHC, was performed using the PRODIGY server. The vaccine + TLR3 docked complex had the binding affinity (ΔG) value of −18.7 kcal mol^−1^. Whereas the vaccine + class I MHC and vaccine + class II MHC docked complexes had binding affinity (ΔG) values of −11.6 kcal mol^−1^ and −16.9 kcal mol^−1^, respectively. The binding affinity values and dissociation constant values for all three dockings are shown in Table 3.

### 3.10. Interacting Residues

**Figure 12:**
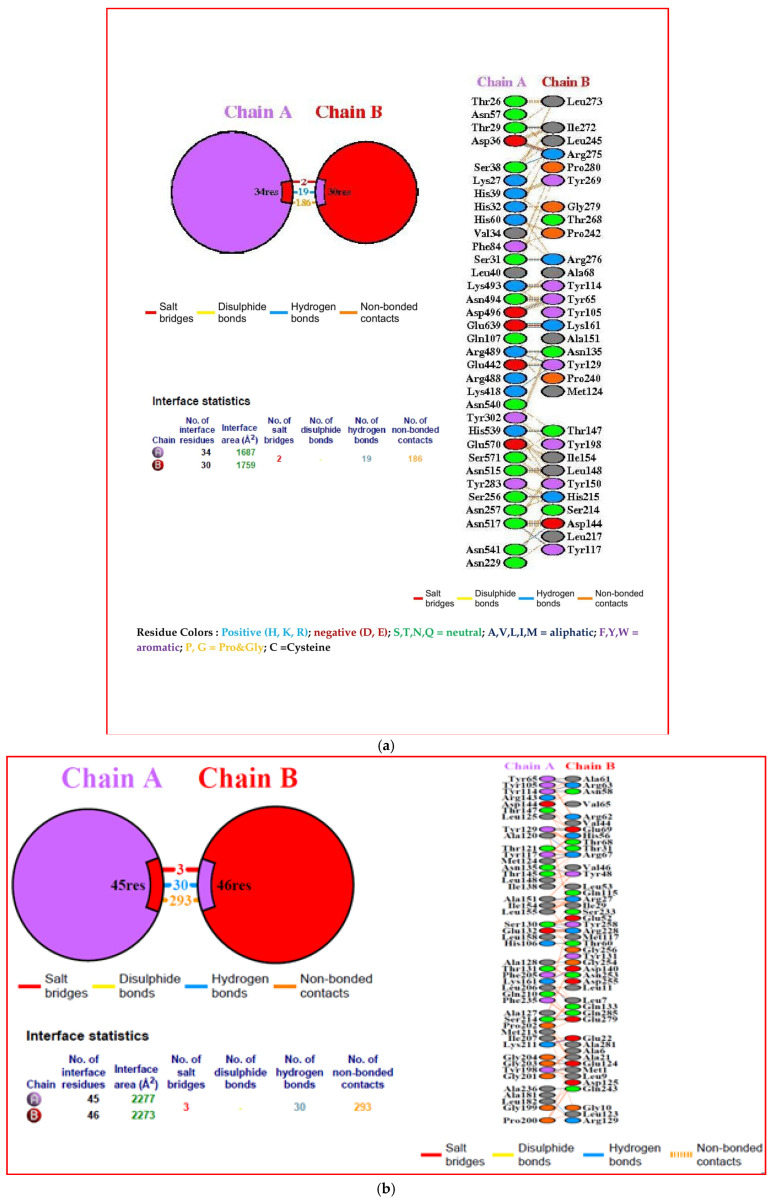

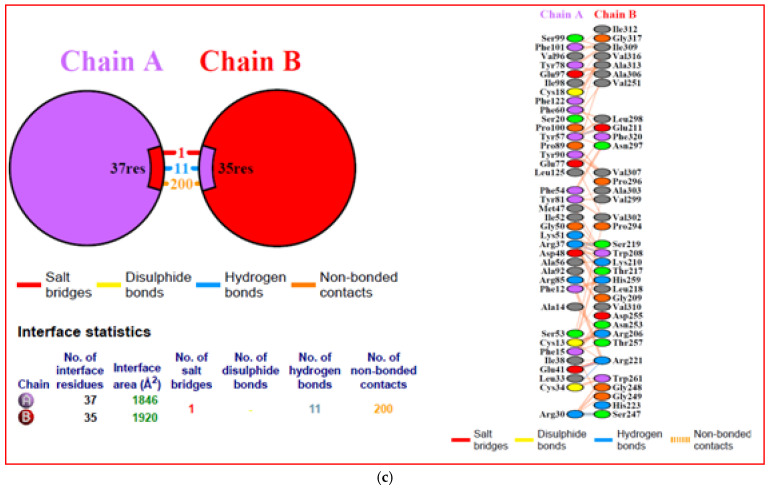
(**a**) Interacting residues between the final multi-epitope vaccine with TLR 3 (**b**), Interacting residues between the final multi-epitope vaccine with class I MHC (**c**),and Interacting residues between the final multi-epitope vaccine with class II MHC.

The interacting residues among the three docked complexes were analyzed using the PDBsum server. The 34 residues of the vaccine have interacted with 30 residues of TLR3, which occupythe interface area of 1687 A^2^ and 1759 A^2^ between them. There were 2 salt bridges, 19 hydrogen bonds, and 186 non-bonded contacts between the vaccine and TLR3. Whereas vaccine and class I MHC had 3 salt bridges, 30 hydrogen bonds, 293 non-bonded contacts, and 45:46 interface residues with an interface area of 2277:2273 A^2^. The vaccine and class II MHC had one salt bridge, 11 hydrogen bonds, 200 non-bonded contacts, and 37:35 interface residues with an interface area of 1846:1920 A^2^ (Figure 12a–c).

### 3.11. Immune Simulation

**Figure 13:**
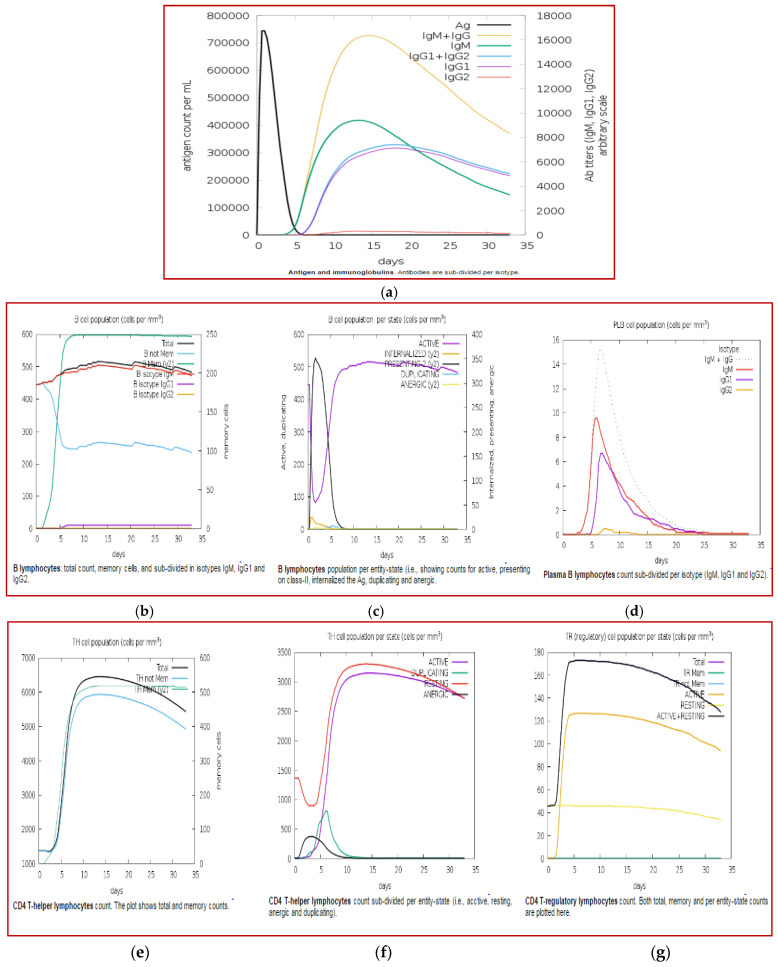

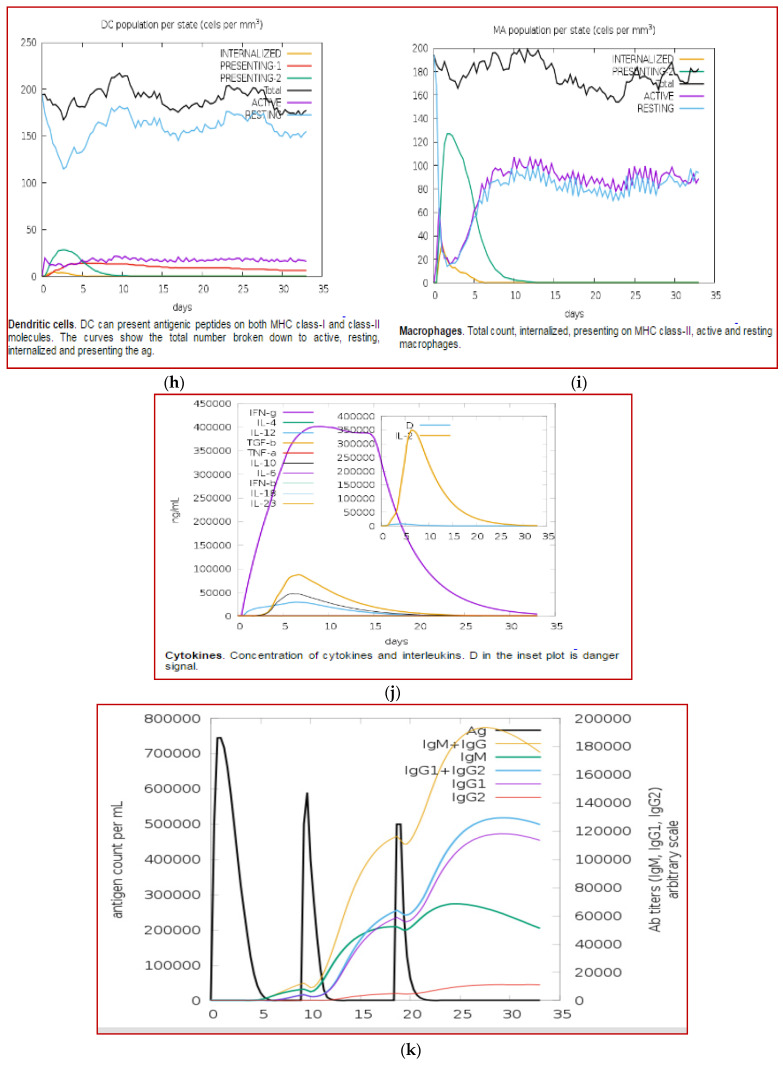

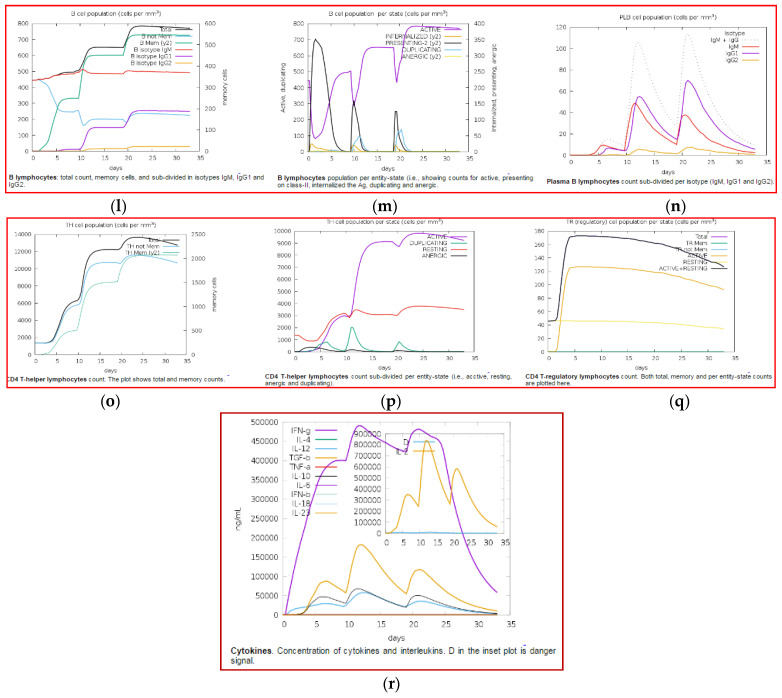
(**a**–**r**) Immune simulation studies of the final vaccine construct.

The immune response profile of the final vaccine was generated using in silico immune simulation assays by the C-IMMSIM server. The immunoglobulins IgM + IgG and IgM were increased during exposure to a single dose of antigen compared to IgG1 + IgG2 and IgG1. The decreasing antigen concentration in secondary and tertiary responses compared to primary generated higher immune responses with increased levels of immunoglobulin activity, such as IgM + IgG, IgG1 + IgG2, IgG1, and IgM. Further, the primary immunoglobulin of chicken is IgY, which is functionally equivalent to IgG in mammals. The immune cell population, such as helper T lymphocytes and cytotoxic T lymphocytes, was found in higher amounts. In addition, the activity of macrophage cells was also induced in higher amounts by the final vaccine design (Figure 13).

### 3.12. Codon Optimization

The final vaccine construct was optimized for its codon usage for protein expression in the prokaryotic system. To achieve maximum expression, the final vaccine construct’s codons were optimized using the Jcat tool and tailored to the expression host system, i.e., the K-12 strain of *E. coli*. The reverse-translated vaccine had a cDNA sequence of 912 nucleotides long after optimizing its codon. The improved sequence had a codon adaptation index (CAI) value of 1.0 and a GC content of 53.61. For effective expression in the host, the CAI value should be greater than 0.8, and the GC content should be from 30% to 70%. The reverse-translated vaccine had a good range of GC content and CAI value that showed efficient expression in a prokaryotic system, the K-12 strain of *E. coli*.

## 4. Discussion

An immunoinformatic approach-based multi-epitope vaccine candidate, comprised of immunogenic T and B cell epitopes, results in the induction of high levels of the protective immune response that lacks reversal of pathogenesis [40]. In this study, ILTV’s glycoproteins B and D were targeted to develop a multi-epitope-based vaccine for ILTV using various bioinformatics tools to produce effective immune responses comprising both humoral and cellular immunity. The reason for targeting these two proteins particularly is that they are major antigenic determinants and also play a vital role in viral adherence and entry into the cell. In addition, these two proteins were shown to be highly conserved and also have virus-neutralization activity [41].

In the present study, we have retrieved amino acid sequences for both glycoproteins B and D of the ILTV using the NCBI database. In the present study, 27 and 10 high-scoring CTL epitopes were filtered for ILTV’s glycoproteins B and D, respectively. CTL epitopes are essentially required to induce cell-mediated immunity, which plays a vital role in protecting chickens against infectious laryngotracheitis viral infection. Cell-mediated immune responses were reported to be the most important arm of responses in ILTV protection in chickens rather than the humoral immune response. Additionally, the CTL epitopes play an essential role in providing long-term adaptive immune responses that lead to the elimination of not only the virus but also virus-infected cells. The 9-mer length CTL epitopes were predicted using the Net CTL server, which has been trained to select only 9-mer length good binding affinity class I MHC ligands. In the present study, six and five high-scoring HTL epitopes were selected for ILTV’s glycoproteins B and D, respectively. The HTL epitopes are crucial for the induction of both cell-mediated and antibody immune responses by stimulating CD8+ and CD4+ T cells, as well as stimulating B cells for the production of antibodies. Similarly, Ali et al.(2019) [42] predicted three CTL and three HTL epitopes using glycoprotein B of ILTV by the immune epitope database. In the recent past, many studies reported the use of HLA alleles instead of chicken MHC sets for the prediction of T cell epitopes to design multi-epitope vaccine candidates against poultry pathogens, viz. Newcastle disease virus [43], Hydropericardium syndrome [44], Chicken infectious anemia [45], *Mycoplasma gallisepticum* [46] and Avian leukosis virus [47].

In the present study, six and five numbers of IFN-γ-inducing epitopes were predicted for ILTV’s glycoproteins B and D, respectively. The IFN-γepitopes play an important role in antiviral, antitumor, and immune regulation mechanisms. Similarly, Pandey et al. (2018) [27] predicted IFN-γinducing epitopes using all HTL epitopes by the IFNepitope server for designing a multi-epitope-based vaccine against Zika virus infection. In the present study, the predicted CTL and HTL epitopes showed high affinity towards class I as well as class II MHC alleles, respectively. These high-affinity epitopes were only used for designing multi-epitope vaccine candidates, which were further subjected to various antigenic, allergenic and toxicity analysis filters. The same approach has been used for the selection of epitopes for designing multi-epitope vaccine candidates against SARS-CoV-2 [48].

The final multi-epitope vaccine candidate was constructed, which comprises four domains, including an adjuvant, CTL, HTL, and His tag. Defensins are potent adjuvants when combined with viral antigens and also play a vital role in triggering both adaptive and innate immune responses in the face of viral infections. The avian β defensin 1 synthetic peptides were demonstrated to be a potent adjuvant in IBDV VP2 DNA vaccines, which results in improved protective efficacy compared to that of the DNA vaccine without avian β defensin [49]. In the present study, chicken β defensin 1, also known as avian β defensin (AvBD1), was added to the N-terminal of the final vaccine candidate as an adjuvant with suitable linkers. Many researchers used the adjuvant human β defensin to enhance the immunogenicity of the final vaccine construct in many multi-epitope vaccines, including Zika virus, Coronavirus, and Kaposi sarcoma virus [27].

The molecular weight should be greater than 10 kDa for the ideal immunogenic nature of the vaccine candidate. The theoretical protrusion index (PI) value for the vaccine construct was 8.87, which indicates the basic nature of our final vaccine design. The half-life and the instability index classified our vaccine candidate as stable. The instability index is calculated for analyzing the stability of the antigen, which should be less than 40 for the vaccine antigen [50]. The aliphatic index was also calculated for our vaccine construct to find out the thermostability of the vaccine antigen. The final vaccine design in the present study had an aliphatic index value of 78.52, which showed the vaccine antigen as thermostable. The thermostability of the vaccine antigen is greater if it has a higher aliphatic index [51]. The GRAVY value was found to be 0.096, which indicates their hydrophobic nature [52]. The antigenic nature of the vaccine construct was found to be 0.617, showing its probable antigenic nature with an above-threshold level of 0.4. Furthermore, the final vaccine construct was also evaluated for its allergenic nature using the AllerTOP server and was shown to be nonallergenic. The mammalian reticulocytes, otherwise known as immature red blood cells, are important for in vitro expression and testing of vaccine constructs during vaccine design. Additionally, these reticulocytes are also used to assess the stability, longevity and immune response studies of vaccine candidates. The anticipated half-life is likely to be about 30h, which shows the resilience of multi-epitope vaccines across different biological environments. The physicochemical properties of the final vaccine design were comparable to those of earlier reports for designing a multi-epitope vaccine against Newcastle disease virus, Chicken infectious anemia, Avian leukosis virus, etc. [27,43,45,47,48].

The obtained 3D model validation results showed the genuineness of the overall quality, reliability, and stability of predicted protein structures. The results for validation of 3D models were consistent with recently published reports of multi-epitope vaccines against Toxoplasmosis [53].

Molecular docking with TLR3 was performed to find out the binding affinity between the final vaccine construct and TLR3. They are pattern recognition receptors that play a central role in the innate immune system, which detects the conserved molecular patterns of pathogens such as viruses that lead to stimulation of innate immunity and also coordinate the adaptive immunity. TLR3 was also reported to recognize DNA viruses such as HSV, Kaposi sarcoma virus and chicken infectious anemia. Recently, the TLR3 structure was used to dock with the multi-epitope vaccine against chicken infectious anemia virus and showed stable interaction by having six hydrogen bonds and low binding energy [45]. Further, the interaction of MHC class I and class II receptors with CTL and HTL epitopes was studied for the generation of cell-mediated as well as humoral immune responses, respectively. The best-docked protein-protein complex was determined on the basis ofa negative energy score. Similarly, Kar et al. (2020) [48] analyzed the molecular docking of the SARS-CoV-2 multi-epitope vaccine design with MHC class I and class II receptors.

The results of binding affinity values showed that all three docked complexes have negative energy values, and all are energetically feasible. The findings were similar to those of an earlier design of a multi-epitope vaccine construct against SARS-CoV-2 [48]. The C-ImmSim model has been reported for the assessment of humoral as well as cell-mediated immune responses for many multi-epitope vaccine constructs. The chicken immune system, like mammals, involves cellular participation like T cells, B cells, and macrophages and uses cytokines and various signaling molecules. Many researchers have already used this model to assess the immune response profile against multi-epitope final vaccine design for various poultry diseases, viz., chicken anemia virus, infectious bursal disease virus, avian influenza, avian leukosis virus, etc. In the present study, the high levels of interferon-gamma and IL-2 were induced during the exposure by immune simulation studies, which showed the effective immune response generated by our vaccine design.

## 5. Conclusions

The final multi-epitope vaccine was designed by using four domains, which compriseavian β defensin 1 sequence as an adjuvant on the N-terminal side, followed by 13 CTL epitopes, five HTL epitopes, including IFN-γ inducing epitopes, as well as B cell epitopes as overlapping epitopes of glycoproteins B and D of ILTV. Immune simulation studies of the final vaccine construct by in-silico method showed an increased amount of both immunoglobulin activity as well as immune cell population, such as helper and cytotoxic T lymphocytes and showed stable interactions with the immune receptors. The positive results of the final multi-epitope vaccine were obtained in the present study by using computational tools. The multi-epitope vaccine depends on short epitopes, which may have altered immunity and require further experimental validations to develop a potential vaccine against ILTV.

## Figures and Tables

**Table 1:** Physiochemical and secondary structure properties of Glycoprotein B and Glycoprotein D.

Target Proteins (Consensus Sequence)		Mol. Wt. (Dalton)		Theoretical pI	Half-Life (in Mammalian Reticulocytes, In Vitro)		Antigeznecity (Threshold 0.4)	
Glycoprotein B		100,174.88		6.38	30 h		0.5010	
Glycoprotein D		48,472.79		5.34	30 h		0.4948	
**Secondary structure properties**								
Glycoprotein B					Glycoprotein D			
Alpha helix	(Hh)		337 is 38.17%		Alpha helix	(Hh)		152 is 35.02%
3_10_helix	(Gg)		0 is 0.00%		3_10_helix	(Gg)		0 is 0.00%
Pi helix	(Ii)		0 is 0.00%		Pi helix	(Ii)		0 is 0.00%
Beta bridge	(Bb)		0 is 0.00%		Beta bridge	(Bb)		0 is 0.00%
Extended Strand	(Ee)		150 is 16.99%		Extended Strand	(Ee)		68 is 15.67%
Beta turn	(Tt)		35 is 3.96%		Beta turn	(Tt)		34 is 7.83%
Bend region	(Ss)		0 is 0.00%		Bend region	(Ss)		0 is 0.00%
Random coil	(Cc)		361 is 40.88%		Random coil	(Cc)		180 is 41.47%
Ambiguous states	(?)		0 is 0.00%		Ambiguous states	(?)		0 is 0.00%
Other states			0 is 0.00%		Other states			0 is 0.00%

**Table 2:** Physiochemical properties of the final multi-epitope vaccine design.

S.No	Particulars	Value
1.	Number of amino acids	304
2.	Molecular weight	32,768.29
3.	Theoretical pI	8.87
4.	Total number of negatively charged residues (Asp + Glu)	16
5.	Total number of positively charged residues (Arg + Lys)	23
6.	Formula	C_1508_H_2233_N_383_O_418_S_11_
7.	Total number of atoms	4553
8.	The estimated half-life is Mammalian reticulocytes-in vitro Yeast-in vivo Escherichia coli-in vivo	30 h >20 h >10 h
9.	Instability index	33.88
10.	Aliphatic index	78.52
11.	Grand average of hydropathicity (GRAVY)	0.096
12.	Solubility	0.423
13.	Antigenicity	0.6177
14.	Allergenicity	Non-allergen

**Table 3:** Binding affinity analysis of the docked complex.

S.No.	Complexes	Binding Affinity (ΔG) (kcal mol^−1^)	Dissociation Constant Kd(M)
1.	TLR 3 + Vaccine construct	−18.7	1.9 × 10^−14^
2.	MHC I + Vaccine construct	−16.9	4.1 × 10^−13^
3.	MHC II + Vaccine construct	−11.6	2.9 × 10^−9^

## Data Availability

The original contributions presented in this study are included in the article/Supplementary Materials; further inquiries can be directed to the corresponding authors.

## Supplementary Materials

The following supporting information can be downloaded at: https://www.mdpi.com/article/10.3390/biology14070765/s1. Supplementary File S1: Title Epitope prediction-ILTV.

## References

[1] García M., Spatz S.J., Guy J.S., Swayne D.E., Glisson J.R., McDougald L.R., Nolan L.K., Suarez D.L., Nair V. (2013). Infectious laryngotracheitis. Diseases of Poultry.

[2] Crawshaw G.J., Boycott B.R. (1982). Infectious laryngotracheitis in peafowl and pheasants. Avian Dis..

[3] Portz C., Beltrão N., Furian T.Q., Júnior A.B., Macagnan M., Griebeler J., Lima Rosa C.A., Colodel E.M., Driemeier D., Back A. (2008). Natural infection of turkeys by infectious laryngotracheitis virus. Vet. Microbiol..

[4] Davison A.J., Eberle R., Hayward G.S., Mcgeoch D.J., Minson A.C., Pellet P.E., Roizman B., Studdert M.J., Thiry E. (2009). The order herpesvirales. Arch. Virol..

[5] Granzow H., Klupp B.G., Fuchs W., Veits J., Osterrieder N., Mettenleiter T.C. (2001). Egress of alphaherpes viruses: Comparative ultrastructural study. J. Virol..

[6] Roziman B., Pellett P.E., Knipe D.M., Howley P.M. (2001). The family Herpesviridae: A brief introduction. Fields Virology.

[7] Meulemans G., Halen P. (1978). Some physicochemical and biological properties of a Belgian strain (U76/1035) of infectious laryngotracheitis virus. Avian Pathol..

[8] Neighbour N.K., Newberry L.A., Bayyari G.R., Skeeles J.K., Beasley J.N., McNew R.W. (1994). The effect of microaerosolized hydrogen peroxide on bacterial and viral pathogens. Poult. Sci..

[9] Islam M.S., Khan M.S.R., Islam M.A., Hassan J. (2010). Isolation and characterization of infectious laryngotracheitis virus in layer chickens. Bangladesh J. Vet. Med..

[10] McGeoch D.J., Dolan A., Ralph A.C. (2000). Toward a comprehensive phylogeny for mammalian and avian herpesviruses. J. Virol..

[11] Morales Ruiz S., Bendezu Eguis J., Montesinos R., Tataje-Lavanda L., Fernández-Díaz M. (2018). Full-genome sequence of infectious laryngotracheitis virus (GallidAlphaherpesvirus 1) strain VFAR-043, isolated in Peru. Genome Announc..

[12] Thureen D.R., Keeler C.L. (2006). Psittacidherpesvirus 1 and infectious laryngotracheitis virus: Comparative genome sequence analysis of two avian alphaherpesviruses. J. Virol..

[13] Lee S.W., Devlin J.M., Markham J.F., Noormohammadi A.H., Browning G.F., Ficorilli N.P., Hartley C.A., Markham P.F. (2011). First complete genome sequence of infectious laryngotracheitis virus. BMC Genom..

[14] Piccirillo A., Lavezzo E., Niero G., Moreno A., Massi P., Franchin E., Toppo S., Salata C., Palù G. (2016). Full genome sequence based comparative study of wild type and vaccine strains of infectious laryngotracheitis virus from Italy. PLoS ONE.

[15] Connolly S.A., Jackson J.O., Jardetzky T.S., Longnecker R. (2011). Fusing structure and function: A structural view of the herpesvirus entry machinery. Nat. Rev. Microbiol..

[16] Zhao W., Spatz S., Zhang Z., Wen G., Garcia M., Zsak L. (2014). Newcastle disease virus (NDV) recombinants expressing infectious laryngotracheitis virus (ILTV)glycoproteins gB and gD protect chickens against ILTV and NDV challenges. J. Virol..

[17] Guy J.S., Barnes H.J., Smith L. (1991). Increased virulence of modified-live infectious laryngotracheitis vaccine virus following bird-to-bird passage. Avian Dis..

[18] Oyarzun P., Ellis J.J., Gonzalez-Galarza F.F., Jones A.R., Middleton D., Boden M., Kobe B. (2015). A bioinformatics tool for epitope based vaccine design that accounts for human ethnic diversity: Application to emerging infectious diseases. Vaccine.

[19] Chauhan V., Rungta T., Goyal K., Singh M.P. (2019). Designing a multi-epitope based vaccine to combat Kaposi Sarcoma utilizing immunoinformatics approach. Sci. Rep..

[20] Wilkins M.R., Gasteiger E., Bairoch A., Sanchez J.C., Williams K.L., Appel R.D., Hochstrasser D.F. (1999). Protein identification and analysis tools in the ExPAsy server. Methods Mol. Biol..

[21] Kallberg M., Wang H., Wang S., Peng J., Wang Z., Lu H., Xu J. (2012). Template based protein structure modelling using the RaptorX web server. Nat. Protoc..

[22] Heo L., Park H., Seok C. (2013). GalaxyRefine: Protein structure refinement driven by side chain repacking. Nucleic Acids Res..

[23] Lovell S.C., Davis I.W., Arendall W.B., Bakker P.I.W.D., Word J.M., Prisant M.G., Richardson J.S., Richardson D.C. (2003). Structure validation by Cαgeometry: ϕ,ψ and Cβdeviation. Proteins Struct. Funct. Bioinform..

[24] Larsen M.V., Lundegaard C., Lamberth K., Buus S., Lund O., Nielsen M. (2007). Large scale validation of methods for cytotoxic T-lymphocyte epitope prediction. BMC Bioinform..

[25] Doytchinova I.A., Flower D.R. (2007). VaxiJen: A server for prediction of protective antigens, tumor antigens and subunit vaccines. BMC Bioinform..

[26] Vita R., Overton J.A., Greenbaum J.A., Ponomarenko J., Clark J.D., Cantrell J.R., Wheeler D.K., Gabbard J.L., Hix D., Sette A. (2015). The immune epitope database (IEDB) 3.0. Nucleic Acids Res..

[27] Pandey R.K., Ojha R., Mishra A., Prajapati V.K. (2018). Designing B and T cell multiepitope based subunit vaccine using immunoinformatics approach to control Zika virus infection. J. Cell. Biochem..

[28] Jensen K.K., Andreatta M., Marcatili P., Buus S., Greenbaum J.A., Yan Z., Sette A., Peters B., Nielsen M. (2018). Improved methods for predicting peptide binding affinity to MHC class II molecules. Immunology.

[29] Dimitrov I., Bangov I., Flower D.R., Doytchinova I. (2014). AllerTOP v.2—A server for in silico prediction of allergens. J. Mol. Model..

[30] Dhanda S.K., Vir P., Raghava G.P. (2013). Designing of interferon gamma inducing MHC class II binders. Biol. Direct..

[31] Arai R., Ueda H., Kitayama A., Kamiya N., Nagamune T. (2001). Design of the linkers which effectively separate domains of a bifunctional fusion protein. Protein Eng..

[32] Cheng J., Randall A.Z., Sweredoski M.J., Baldi P. (2005). SCRATCH: A protein structure and structural feature prediction server. NucleicAcids Res..

[33] Wiederstein M., Sippl M.J. (2007). ProSA-web: Interactive web service for the recognition of errors in three dimensional structures of proteins. Nucleic Acids Res..

[34] Colovos C., Yeates T.O. (1993). Verification of protein structures: Patterns of nonbonded atomic interactions. Protein Sci..

[35] Vangone A., Bonvin A.M. (2015). Contacts based prediction of binding affinity in protein-protein complexes. eLife.

[36] Xue L.C., Rodrigues J.P., Kastritis P.L., Bonvin A.M., Vangone A. (2016). PRODIGY: A web server for predicting the binding affinity of protein-protein complexes. Bioinformatics.

[37] Laskowski R.A., Jablonska J., Pravda L., Varekova R.S., Thornton J.M. (2018). PDBsum: Structural summaries of PDB entries. Protein Sci..

[38] Rapin N., Lund O., Bernaschi M., Castiglione F. (2010). Computational immunology meets bioinformatics: The use of prediction tools for molecular binding in the simulation of the immune system. PLoS ONE.

[39] Grote A., Hiller K., Scheer M., Munch R., Nortemann B., Hempel D.C., Jahn D. (2005). JCat: A novel tool to adapt codon usage of a target gene to its potential expression host. Nucleic Acids Res..

[40] Zhang L. (2018). Multi-epitope vaccines: A promising strategy against tumors and viral infections. Cell. Mol. Immunol..

[41] KanabagatteBasavarajappa M., Song H., Lamichhane C., Samal S.K. (2015). Glycoprotein based enzyme-linked immunosorbent assays for serodiagnosis of infectious laryngotracheitis. J. Clin. Microbiol..

[42] Ali S.A., Almofti Y.A., Abd-Elrahman K.A. (2019). Immunoinformatics approach for multiepitopes vaccine prediction against Glycoprotein B of Avian infectious laryngotracheitis virus. Adv. Bioinform..

[43] Hosseini S.S., Kolyani K.A., Tabatabaei R.R., Goudarzi H., Sepahi A.A., Salemi M. (2021). In silico prediction of B and T cell epitopes based on NDV fusion protein for vaccine development against Newcastle disease virus. Vet. Res. Forum..

[44] Aziz F., Tufail S., Shah M.A., Salahuddin Shah M., Habib M., Mirza O., Iqbal M., Rahman M. (2019). In Silico epitope prediction and immunogenic analysis for penton base epitope-focused vaccine against Hydropericardium Syndrome in Chicken. Virus Res..

[45] Fatoba A.J., Adeleke V.T., Maharaj L., Okpeku M., Adeniyi A.A., Adeleke M.A. (2022). Design of a multiepitope vaccine against Chicken Anemia virus disease. Viruses.

[46] Mugunthan S.P., Mani Chandra H. (2021). A computational reverse vaccinology approach for the design and development of multi-epitopic vaccine against avian pathogen *Mycoplasma gallisepticum*. Front. Vet. Sci..

[47] Elshafei S.O., Mahmoud N.A., Almofti Y.A. (2024). Immunoinformatics, molecular docking and dynamics simulation approaches unveil a multi epitope-based potent peptide vaccine candidate against avian leukosis virus. Sci. Rep..

[48] Kar T., Narsaria U., Basak S., Deb D., Castiglione F., Mueller D.M., Srivastava A.P. (2020). A candidate multi-epitope vaccine against SARS-CoV-2. Sci. Rep..

[49] Zhang H.H., Yang X.M., Xie Q.M., Ma J.Y., Luo Y.N., Cao Y.C., Chen F., Bi Y.Z. (2010). The potent adjuvant effects of chicken beta-defensin-1 when genetically fused with infectious bursal disease virus VP2 gene. Vet. Immunol. Immunopathol..

[50] Walker J.M. (2005). The Proteomics Protocols Handbook.

[51] Ikai A. (1980). Thermostability and aliphatic index of globular proteins. J. Biochem..

[52] Kyte J., Doolitttle R.F. (1982). A simple method for displaying the hydropathic character of a protein. J. Mol. Biol..

[53] Foroutan M., Ghaffaifar F., Sharifi Z., Dalimi A. (2020). Vaccination with a novel multi-epitope ROP8 DNA vaccine against acute *Toxoplasma gondii* infection induces strong B and T cell responses in mice. Comp. Immunol. Microbiol. Infect. Dis..

