# Supplementary material for: Design of a Multi-Epitope Vaccine Candidate Against Infectious Laryngotracheitis Virus Affecting Poultry by Computational Approaches
Source: Biology (Basel). 2025 Jun 25;14(7):765. doi: 10.3390/biology14070765 (PMC12292712; doi:10.3390/biology14070765)
Supplement: Supplementary file 1 [file biology-14-00765-s001.zip › Epitope prediction -ILT.pdf]

Prediction of B and T cell epitopes of immunogenic glycoproteins B and D of ILT virus

Retrieval of amino acid sequences of the Glycoprotein B of ILT Virus

A total of 67 numbers of amino acid sequences of Glycoprotein B of ILT Virus were retrieved by utilizing NCBI database. The retrieved amino acid sequences were subjected into multiple sequence alignment to find out the conservancy among the proteins. Further, the consensus sequence was created for Glycoprotein B of ILT virus by using Bioedit software.

Target glycoproteins	Accession numbers (retrieved from NCBI Database)
Glycoprotein B	ABX59525.1 envelope glycoprotein B [Gallid alphaherpesvirus 1]<br>ABX59524.1 envelope glycoprotein B [Gallid alphaherpesvirus 1]<br>AUT11958.1 envelope glycoprotein B [Gallid alphaherpesvirus 1]<br>YP_182356.1 envelope glycoprotein B [Gallid alphaherpesvirus 1]<br>AWL83342.1 envelope glycoprotein B [Gallid alphaherpesvirus 1]<br>AWL83263.1 envelope glycoprotein B [Gallid alphaherpesvirus 1]<br>ATG31674.1 envelope glycoprotein B [Gallid alphaherpesvirus 1]<br>ATG31596.1 envelope glycoprotein B [Gallid alphaherpesvirus 1]<br>ATG31518.1 envelope glycoprotein B [Gallid alphaherpesvirus 1]<br>ATG31440.1 envelope glycoprotein B [Gallid alphaherpesvirus 1]<br>ATG31362.1 envelope glycoprotein B [Gallid alphaherpesvirus 1]<br>ATD84531.1 envelope glycoprotein B [Gallid alphaherpesvirus 1]<br>ATD84452.1 envelope glycoprotein B [Gallid alphaherpesvirus 1]<br>ATD84373.1 envelope glycoprotein B [Gallid alphaherpesvirus 1]<br>ATD84294.1 envelope glycoprotein B [Gallid alphaherpesvirus 1]<br>ATD84215.1 envelope glycoprotein B [Gallid alphaherpesvirus 1]<br>ATD84136.1 envelope glycoprotein B [Gallid alphaherpesvirus 1]<br>ANB43607.1 envelope glycoprotein B [Gallid alphaherpesvirus 1]<br>ANF04484.1 envelope glycoprotein B [Gallid alphaherpesvirus 1]<br>ANN24991.1 envelope glycoprotein B [Gallid alphaherpesvirus 1]<br>ANN24921.1 envelope glycoprotein B [Gallid alphaherpesvirus 1]<br>AJR27653.1 envelope glycoprotein B [Gallid alphaherpesvirus 1]<br>AJR27811.1 envelope glycoprotein B [Gallid alphaherpesvirus 1]<br>AJR27732.1 envelope glycoprotein B [Gallid alphaherpesvirus 1]<br>AJR27574.1 envelope glycoprotein B [Gallid alphaherpesvirus 1]<br>AJR27495.1 envelope glycoprotein B [Gallid alphaherpesvirus 1]<br>AER28131.1 envelope glycoprotein B [Gallid alphaherpesvirus 1]<br>AER28052.1 envelope glycoprotein B [Gallid alphaherpesvirus 1]<br>AGN48336.1 envelope glycoprotein B [Gallid alphaherpesvirus 1]<br>AGN48256.1 envelope glycoprotein B [Gallid alphaherpesvirus 1]<br>AGN48178.1 envelope glycoprotein B [Gallid alphaherpesvirus 1]<br>AGC23137.1 envelope glycoprotein B [Gallid alphaherpesvirus 1]<br>AGC23058.1 envelope glycoprotein B [Gallid alphaherpesvirus 1]<br>AFN02008.1 envelope glycoprotein B [Gallid alphaherpesvirus 1]<br>AFN01929.1 envelope glycoprotein B [Gallid alphaherpesvirus 1]<br>AEW67850.1 envelope glycoprotein B [Gallid alphaherpesvirus 1]<br>AEW67771.1 envelope glycoprotein B [Gallid alphaherpesvirus 1]

	AEB97319.1 envelope glycoprotein B [Gallid alphaherpesvirus 1]<br>ABX59533.1 envelope glycoprotein B [Gallid alphaherpesvirus 1]<br>ABX59532.1 envelope glycoprotein B [Gallid alphaherpesvirus 1]<br>ABX59531.1 envelope glycoprotein B [Gallid alphaherpesvirus 1]<br>ABX59530.1 envelope glycoprotein B [Gallid alphaherpesvirus 1]<br>ABX59529.1 envelope glycoprotein B [Gallid alphaherpesvirus 1]<br>ABX59528.1 envelope glycoprotein B [Gallid alphaherpesvirus 1]<br>ABX59526.1 envelope glycoprotein B [Gallid alphaherpesvirus 1]<br>ABX59521.1 envelope glycoprotein B [Gallid alphaherpesvirus 1]<br>ABX59520.1 envelope glycoprotein B [Gallid alphaherpesvirus 1]<br>ABX59519.1 envelope glycoprotein B [Gallid alphaherpesvirus 1]<br>ABX59518.1 envelope glycoprotein B [Gallid alphaherpesvirus 1]<br>ABX59517.1 envelope glycoprotein B [Gallid alphaherpesvirus 1]<br>ABX59516.1 envelope glycoprotein B [Gallid alphaherpesvirus 1]<br>ABX59515.1 envelope glycoprotein B [Gallid alphaherpesvirus 1]<br>ABX59514.1 envelope glycoprotein B [Gallid alphaherpesvirus 1]<br>ABX59513.1 envelope glycoprotein B [Gallid alphaherpesvirus 1]<br>ABX59512.1 envelope glycoprotein B [Gallid alphaherpesvirus 1]<br>QHW06654.1 envelope glycoprotein B [Gallid alphaherpesvirus 1]<br>QHW06575.1 envelope glycoprotein B [Gallid alphaherpesvirus 1]<br>QHW06496.1 envelope glycoprotein B [Gallid alphaherpesvirus 1]<br>QHW06418.1 envelope glycoprotein B [Gallid alphaherpesvirus 1]<br>QHW06339.1 envelope glycoprotein B [Gallid alphaherpesvirus 1]<br>QHW06260.1 envelope glycoprotein B [Gallid alphaherpesvirus 1]<br>QHW06182.1 envelope glycoprotein B [Gallid alphaherpesvirus 1]<br>QHW06103.1 envelope glycoprotein B [Gallid alphaherpesvirus 1]<br>QGA87041.1 envelope glycoprotein B [Gallid alphaherpesvirus 1]<br>QAA96215.1 envelope glycoprotein B [Gallid alphaherpesvirus 1]<br>QAA96137.1 envelope glycoprotein B [Gallid alphaherpesvirus 1]<br>QAA96059.1 envelope glycoprotein B [Gallid alphaherpesvirus 1]

The Consensus sequence of Glycoprotein B of ILT Virus

MQSYIAVNIDMASLKMLICVCVAILIPSTLSQDSHGIAGIIDPRDTASMDVGKISFSEAIGSGAPKEP
QIRNRIFACSSPTGASVARLAQPRHCHRHADSTNMTEGIAVVFKQNIAPYVFNVTLYYKHITTVT
TWALFSRPQITNEYVTRVPIDYHEIVRIDRSGECSSKATYHKNFMFFEAYDNDEAEKKLPLVPSLL
RSTVSKAFHTTNFTKRHQTLGYRTSTSVDCVVEYLQARSVYPYDYFGMATGDTVEISPFYTKNT
TGPRRHSVYRDYRFLEIANYQVRDLETGQIRPPKKRNFLTDEQFTIGWDAMEEKESVCTLSKWIE
VPEAVRVSYKNSYHFSLKDMTMTFSSGKQPFNISRLHLAECVPTIATEAIDGIFARKYSSTHVRSG
DIEYYLGSGGFLIAFQKLMSHGLAEMYLEEAQRQNHLPRGRERRQAAGRRTASLQSGPQGDRIT
THSSATFAMLQFAYDKIQAHVNELIGNLLEAWCELQNRQLIVWHEMKKLNPNSLMTSLFGQPVS
ARLLGDIVAVSKCIEIPIENIRMQDSMRMPGDPTMCYTRPVLIFRYSSSPESQFSANSTENHNLDIL
GQLGEHNEILQGRNLIEPCMINHRRYFLLGENYLLYEDYTFVRQVNASEIEEVSIFINLNATILEDL
DFVPVEVYTREELRDTGTLNYDDVVRYQNIYNKRFRDIDTVIRGDRGDAIFRAIADFFGNTLGEV
GKALGTVVMTAAAAVISTVSGIASFLSNPFAALGIGIAVVVSIILGLLAFKYVMNLKSNPVQVLFP
GAVPPAGTPPRPSRRYYKDEEEVEEDSDEDDRILATRVLKGLELLHKDEQKARRQKARFSAFAK
NMRNLFRRKPRTKEDDYPLLEYPSWAEESEDE

Retrieval of amino acid sequences of the Glycoprotein D of ILT Virus

A total of 56 numbers of amino acid sequences of Glycoprotein D of ILT Virus were retrieved by utilizing NCBI database. The retrieved amino acid sequences were subjected into multiple sequence alignment to find out the conservancy among the proteins. Further, the consensus sequence was created for Glycoprotein D of ILT virus by using Bioedit software.

Target glycoproteins	Accession numbers (retrieved from NCBI Database)
Glycoprotein D	AGS36508.1 glycoprotein D [Gallid alphaherpesvirus 1]<br>AGS36507.1 glycoprotein D [Gallid alphaherpesvirus 1]<br>AFC98769.1 glycoprotein D [Gallid alphaherpesvirus 1]<br>AAC55100.1 glycoprotein D [Gallid alphaherpesvirus 1]<br>ABH03551.1 glycoprotein D [Gallid alphaherpesvirus 1]<br>ABH03552.1 glycoprotein D [Gallid alphaherpesvirus 1]<br>ASR80994.1 glycoprotein D [Gallid alphaherpesvirus 1]<br>QHN60337.1 glycoprotein D [Gallid alphaherpesvirus 1]<br>QHN60336.1 glycoprotein D [Gallid alphaherpesvirus 1]<br>QHN60335.1 glycoprotein D [Gallid alphaherpesvirus 1]<br>QHN60334.1 glycoprotein D [Gallid alphaherpesvirus 1]<br>QHN60333.1 glycoprotein D [Gallid alphaherpesvirus 1]<br>ATG31669.1 envelope glycoprotein D [Gallid alphaherpesvirus 1]<br>ATG31591.1 envelope glycoprotein D [Gallid alphaherpesvirus 1]<br>ATG31513.1 envelope glycoprotein D [Gallid alphaherpesvirus 1]<br>ATG31435.1 envelope glycoprotein D [Gallid alphaherpesvirus 1]<br>ATG31357.1 envelope glycoprotein D [Gallid alphaherpesvirus 1]<br>ANF04508.1 envelope glycoprotein D [Gallid alphaherpesvirus 1]<br>ANN25023.1 envelope glycoprotein D [Gallid alphaherpesvirus 1]<br>ANN24916.1 envelope glycoprotein D [Gallid alphaherpesvirus 1]<br>AJR27860.1 envelope glycoprotein D [Gallid alphaherpesvirus 1]<br>AJR27781.1 envelope glycoprotein D [Gallid alphaherpesvirus 1]<br>AJR27702.1 envelope glycoprotein D [Gallid alphaherpesvirus 1]<br>AJR27623.1 envelope glycoprotein D [Gallid alphaherpesvirus 1]<br>AJR27544.1 envelope glycoprotein D [Gallid alphaherpesvirus 1]<br>AER28180.1 envelope glycoprotein D [Gallid alphaherpesvirus 1]<br>AER28101.1 envelope glycoprotein D [Gallid alphaherpesvirus 1]<br>AGC23183.1 envelope glycoprotein D [Gallid alphaherpesvirus 1]<br>AGC23102.1 envelope glycoprotein D [Gallid alphaherpesvirus 1]<br>AFN02057.1 envelope glycoprotein D [Gallid alphaherpesvirus 1]<br>AFN01978.1 envelope glycoprotein D [Gallid alphaherpesvirus 1]<br>AEW67899.1 envelope glycoprotein D [Gallid alphaherpesvirus 1]<br>AEW67820.1 envelope glycoprotein D [Gallid alphaherpesvirus 1]<br>AEB97368.1 envelope glycoprotein D [Gallid alphaherpesvirus 1]<br>AUT12004.1 envelope glycoprotein D [Gallid alphaherpesvirus 1]<br>AWL83391.1 envelope glycoprotein D [Gallid alphaherpesvirus 1]<br>AWL83312.1 envelope glycoprotein D [Gallid alphaherpesvirus 1]<br>ATD84554.1 envelope glycoprotein D [Gallid alphaherpesvirus 1]

	ATD84475.1 envelope glycoprotein D [Gallid alphaherpesvirus 1]<br>ATD84396.1 envelope glycoprotein D [Gallid alphaherpesvirus 1]<br>ATD84318.1 envelope glycoprotein D [Gallid alphaherpesvirus 1]<br>ATD84238.1 envelope glycoprotein D [Gallid alphaherpesvirus 1]<br>ATD84159.1 envelope glycoprotein D [Gallid alphaherpesvirus 1]<br>ANB43656.1 envelope glycoprotein D [Gallid alphaherpesvirus 1]<br>QHW06678.1 envelope glycoprotein D [Gallid alphaherpesvirus 1]<br>QHW06599.1 envelope glycoprotein D [Gallid alphaherpesvirus 1]<br>QHW06520.1 envelope glycoprotein D [Gallid alphaherpesvirus 1]<br>QHW06441.1 envelope glycoprotein D [Gallid alphaherpesvirus 1]<br>QHW06363.1 envelope glycoprotein D [Gallid alphaherpesvirus 1]<br>QHW06284.1 envelope glycoprotein D [Gallid alphaherpesvirus 1]<br>QAA96083.1 envelope glycoprotein D [Gallid alphaherpesvirus 1]<br>QAA96161.1 envelope glycoprotein D [Gallid alphaherpesvirus 1]<br>QAA96239.1 envelope glycoprotein D [Gallid alphaherpesvirus 1]<br>QGA87065.1 envelope glycoprotein D [Gallid alphaherpesvirus 1]<br>QHW06127.1 envelope glycoprotein D [Gallid alphaherpesvirus 1]<br>QHW06205.1 envelope glycoprotein D [Gallid alphaherpesvirus 1]

The Consensus sequence of Glycoprotein D of ILT Virus

MHRPHLRRHSRYYAKGEVLNKHMDCGGKRCCSGAAVFTLFWTCVRIMREHICFVRNAMDRHL
FLRNAFWTIVLLSSFASQSTAAVTYDYILGRRALDALTIPAVGPYNRYLTRVSRGCDVVELNPISN
VDDMISAAKEKEKGGPFEASVVWFYVIKGDDGEDKYCPIYRKEYRECGDVQLLSECAVQSAQM
WAVDYVPSTLVSRNGAGLTIFSPTAALSGQYLLTLKIGRFAQTALVTLEVNDRCLKIGSQLNFLPS
KCWTTEQYQTGFQGEHLYPIADTNTRHADDVYRGYEDILQRWNNLLRKKNPSAPDPRPDSVPQ
EIPAVTKKAEGRTPDAESSEKKAPPEDSEDDMQAEASGENPAALPEDDEVPEDTEHDDPNSDPD
YYNDMPAVIPVEETTKSSNAVSMPIFAAFVACAVALVGLLVWSIVKCARS

The details of the homology modeling carried out for the target proteins using different tools

Target proteins	Homology modeling tools employed	Template	Ramachandran Plot analysis
gB	I Tassser		Favoured region:<br>Allowed region:<br>Outlier region:
	Raptor X	3nwfA<br>4bomA<br>6escA	Favoured region: 94.8%)<br>Allowed region: 3.8%<br>Outlier region: 1.4%
	Phyre 2	C5v2sA	Favoured region: 93.3%<br>Allowed region: 4.9%<br>Outlier region: 1.9%
gD	I Tassser	2c36A	Favoured region: 72.4%<br>Allowed region: 15.1%<br>Outlier region:12.5%
	Raptor X		Favoured region:

			Allowed region:<br>Outlier region:
	Phyre 2	c2c36B	Favoured region: 87.3%<br>Allowed region: 8.2%<br>Outlier region: 4.5%

ILT virus protein	Position	CTL Epitopes	MHC Affinity	Cleavage	TAP	Combined Score
Glycoprotein B	413	MSHGLAEMY	0.5065	0.891	3.09	2.439
	617	LLGENYLLY	0.4763	0.779	2.68	2.273
	118	YVFNVTLYY	0.4629	0.968	3.01	2.261
	669	LRDTGTLNY	0.3899	0.963	2.79	1.939
	580	STENHNLDI	0.3631	0.82	0.56	1.693
	225	TSVDCVVEY	0.3144	0.934	3.08	1.629
	251	DTVEISPFY	0.3053	0.957	2.64	1.571
	374	ATEAIDGIF	0.3019	0.462	2.52	1.477
	147	YVTRVPIDY	0.2564	0.908	2.84	1.367
	103	MTEGIAVVF	0.2424	0.797	2.56	1.277
	513	MTSLFGQPV	0.1971	0.939	0.38	0.997
	130	TTVTTWALF	0.169	0.888	2.58	0.98
	461	SSATFAMLQ	0.2287	0.036	0.02	0.976
	390	HVRSGDIEY	0.1515	0.973	3.03	0.941
	48	SMDVGKISF	0.1404	0.97	2.69	0.876
	234	LQARSVYPY	0.1302	0.828	2.97	0.825
	226	SVDCVVEYL	0.1481	0.967	0.98	0.823
	568	YSSSPESQF	0.1286	0.929	2.68	0.819
	460	HSSATFAML	0.1462	0.967	0.91	0.811
	765	ILGLLAFKY	0.1231	0.966	2.81	0.808
	679	DVVRYQNIY	0.1249	0.953	2.69	0.808
	778	KSNPVQVLF	0.1235	0.926	2.64	0.795
	171	ATYHKNFMF	0.1194	0.901	2.95	0.789
	457	ITTHSSATF	0.1236	0.84	2.63	0.782
	865	EDDYPLLEY	0.123	0.943	2.34	0.781
	129	ITTVTTWAL	0.1337	0.965	0.9	0.757
	143	ITNEYVTRV	0.1427	0.938	0.17	0.755

S.No.	ILT virus protein	Position	CTL Epitopes	Antigenicity	Toxicity	Conservancy
1.	Glycoprotein B	413	MSHGLAEMY	0.209	Non-Toxin	100.00 %
		617	LLGENYLLY	-0.154	Non-Toxin	100.00%
		118	YVFNVTLYY	**0.416***	Non-Toxin	100.00%
		669	LRDTGTLNY	**0.814***	Non-Toxin	100.00%
		580	STENHNLDI	**1.253***	Non-Toxin	100.00%
		225	TSVDCVVEY	**0.442***	Non-Toxin	100.00%
		251	DTVEISPFY	**1.020***	Non-Toxin	100.00%
		374	ATEAIDGIF	0.086	Non-Toxin	100.00%
		147	YVTRVPIDY	0.298	Non-Toxin	100.00%
		103	MTEGIAVVF	0.385	Non-Toxin	100.00%
		513	MTSLFGQPV	-0.379	Non-Toxin	100.00%
		130	TTVTTWALF	0.3223	Non-Toxin	100.00%
		461	SSATFAMLQ	**0.609***	Non-Toxin	100.00%
		390	HVRSGDIEY	**2.330***	Non-Toxin	100.00%
		48	SMDVGKISF	**1.393***	Non-Toxin	100.00%
		234	LQARSVYPY	**1.107***	Non-Toxin	100.00%
		226	SVDCVVEYL	0.211	Non-Toxin	100.00%
		568	YSSSPESQF	0.157	Non-Toxin	100.00%
		460	HSSATFAML	**0.646***	Non-Toxin	100.00%
		765	ILGLLAFKY	**1.522***	Non-Toxin	100.00%
		679	DVVRYQNIY	0.188	Non-Toxin	100.00%
		778	KSNPVQVLF	**0.452***	Non-Toxin	100.00%
		171	ATYHKNFMF	**1.055***	Non-Toxin	100.00%
		457	ITTHSSATF	0.264	Non-Toxin	100.00%
		865	EDDYPLLEY	**0.488***	Non-Toxin	100.00%
		129	ITTVTTWAL	**0.456***	Non-Toxin	100.00%
		143	ITNEYVTRV	0.008	Non-Toxin	100.00%

***Antigenic**

S.No	Peptide start	Peptide end	CTL Epitopes	percentile rank	Allele
1	746	754	FLSNPFAAL	0.28	HLA-A*02:03
				0.46	HLA-A*02:06
				0.7	HLA-A*02:01
				0.77	HLA-B*08:01
2	743	751	IASFLSNPF	0.4	HLA-A*32:01
				0.4	HLA-B*35:01

				0.5	HLA-B*15:01
				0.9	HLA-B*53:01
3	778	786	KSNPVQVLF	0.1	HLA-B*58:01
				0.18	HLA-B*57:01
				0.3	HLA-A*32:01
4	247	255	MATGDTVEI	0.2	HLA-B*53:01
				0.8	HLA-B*51:01
				0.8	HLA-A*68:02
				0.8	HLA-B*58:01
5	16	24	MLICVCVAI	0.5	HLA-A*02:01
				0.51	HLA-A*02:03
				0.71	HLA-A*02:06
				0.8	HLA-A*68:02
				0.8	HLA-A*32:01
6	348	356	MTMTFSSGK	0.11	HLA-A*68:01
				0.11	HLA-A*11:01
				0.11	HLA-A*03:01
				0.7	HLA-A*30:01
7	114	122	NIAPYVFNV	0.3	HLA-A*68:02
				0.39	HLA-A*02:06
				0.49	HLA-A*02:03
				0.6	HLA-A*02:01
8	819	827	RILATRVLK	0.13	HLA-A*03:01
				0.23	HLA-A*11:01
				0.5	HLA-A*30:01
				0.65	HLA-A*31:01
9	738	746	STVSGIASF	0.16	HLA-A*26:01
				0.4	HLA-B*15:01
				0.7	HLA-A*32:01
				0.9	HLA-B*58:01
10	370	378	VPTIATEAI	0.2	HLA-B*51:01
				0.7	HLA-B*53:01
				0.7	HLA-B*07:02
				0.76	HLA-B*35:01
11	335	343	VSYKNSYHF	0.35	HLA-A*23:01
				0.5	HLA-B*58:01
				0.64	HLA-B*57:01
				0.7	HLA-B*15:01
12	118	126	YVFNVTLYY	0.11	HLA-A*26:01
				0.23	HLA-A*01:01
				0.26	HLA-A*11:01

				0.4	HLA-B*35:01
				0.41	HLA-A*03:01
				0.5	HLA-B*15:01
				0.58	HLA-A*68:01
				0.83	HLA-A*30:02

Position	Peptide Sequence	Core peptide	Antigenicity	Toxicity	Conservancy
612	HRRYFLLGENYLLYE	FLLGENYLL	**0.425***	Non-Toxin	100.00%
768	LLAFKYVMNLKSNPV	FKYVMNLKS	**0.9508***	Non-Toxin	100.00%
771	FKYVMNLKSNPVQVL	VMNLKSNPV	**0.5747***	Non-Toxin	100.00%
404	GFLIAFQKLMSHGLA	FQKLMSHGL	0.1105	Non-Toxin	100.00%
740	VSGIASFLSNPFAAL	IASFLSNPF	-0.0343	Non-Toxin	100.00%
403	GGFLIAFQKLMSHGL	LIAFQKLMS	0.0161	Non-Toxin	100.00%

***Antigenic**

Interferon (IFN- γ) epitope prediction

The interferon gamma inducing HTL epitopes were predicted by using IFNepitope server. All HTL epitopes were used as an input to identify the presence of the IFN gamma epitope.

Serial No.	Epitope Name	Sequence	Method	Result	Score
1	Epitope_1	HRRYFLLGENYLLYE	SVM	POSITIVE	0.39919982
2	Epitope_2	LLAFKYVMNLKSNPV	SVM	NEGATIVE	-0.63490103
3	Epitope_3	FKYVMNLKSNPVQVL	SVM	NEGATIVE	-0.57721738
4	Epitope_4	GFLIAFQKLMSHGLA	SVM	POSITIVE	0.33887961
5	Epitope_5	VSGIASFLSNPFAAL	SVM	POSITIVE	0.060045525
6	Epitope_6	GGFLIAFQKLMSHGL	SVM	POSITIVE	0.21454116

B cell epitopes identification

Linear B cell epitopes

The linear/continuous B cell epitopes were identified using IEDB. Further the predicted B cell epitopes were also assessed for surface accessibility and antigenicity.

Prediction of B cell epitopes by Emni surface accessibility

No.	Start	End	Predicted Peptide	Length
1	64	71	APKEPQIR	8
2	97	103	HADSTNM	7
3	170	175	KATYHK	6
4	182	191	AYDNDEAEKK	10

5	210	217	TNFTKRHQ	8
6	258	276	FYTKNTTGPRRHSVYRDYR	19
7	291	301	GQIRPPKKRNF	11
8	336	341	SYKNSY	6
9	423	446	EEAQRQNHLPRGRERRQAAGRRTA	24
10	503	508	EMKKLN	6
11	543	549	IRMQDSM	7
12	568	574	YSSSPES	7
13	579	584	NSTENH	6
14	664	672	YTREELRDT	9
15	684	692	QNIYNKRFR	9
16	793	818	AGTPPRPSRRYYKDEEEVEEDSDEDD	26
17	832	844	LHKDEQKARRQKA	13
18	856	867	LFRRKPRTKEDD	12

Prediction of B cell epitopes by Emni surface accessibility

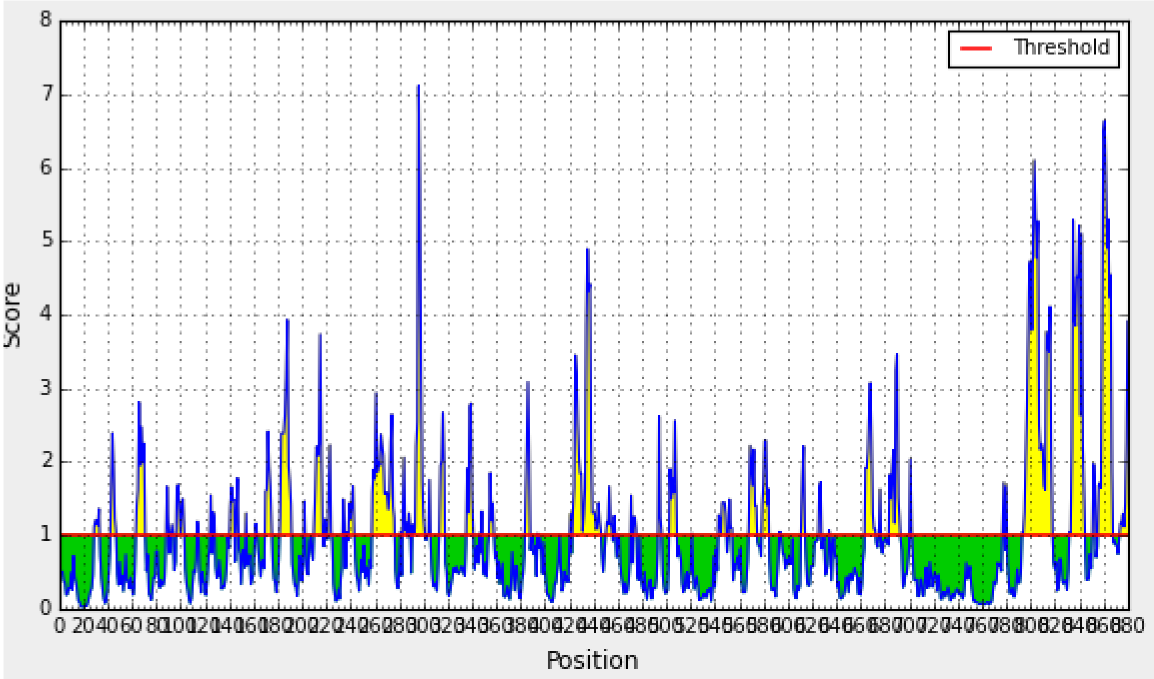

*Regions above redline (threshold) are proposed to be part of B cell epitope

Prediction of B cell epitopes by Kolaskar and Tongaonkar antigenicity prediction

No.	Start	End	Predicted Peptide	Length
1	15	29	KMLICVCVAILIPST	15
2	75	80	FACSSP	6
3	91	97	PRHCHRH	7
4	107	113	IAVVFKQ	7
5	116	130	APYVFNVTLYYKHIT	15

6	148	159	VTRVPIDYHEIV	12
7	191	202	KLPLVPSLLRST	12
8	226	234	SVDCVVEYL	9
9	236	243	ARSVYPYD	8
10	318	324	ESVCTLS	7
11	330	336	PEAVRVS	7
12	363	373	RLHLAECVPTI	11
13	405	410	FLIAFQ	6
14	475	481	IQAHVNE	7
15	496	501	RQLIVW	6
16	518	524	GQPVSAR	7
17	526	538	LGDIVAVSKCIEI	13
18	560	568	TRPVLIFRY	9
19	620	628	ENYLLYEDY	9
20	655	664	DLDFVPVEVY	10
21	678	684	DDVVRYQ	7
22	724	746	ALGTVVMTAAAAVISTVSGIASF	23
23	753	772	ALGIGIAVVVSIILGLLAFK	20
24	780	793	NPVQVLFPGAVPPA	14
25	826	832	LKGLELL	7
26	868	874	YPLLEYP	7

Prediction of B cell epitopes by Kolaskar and Tongaonkar antigenicity prediction

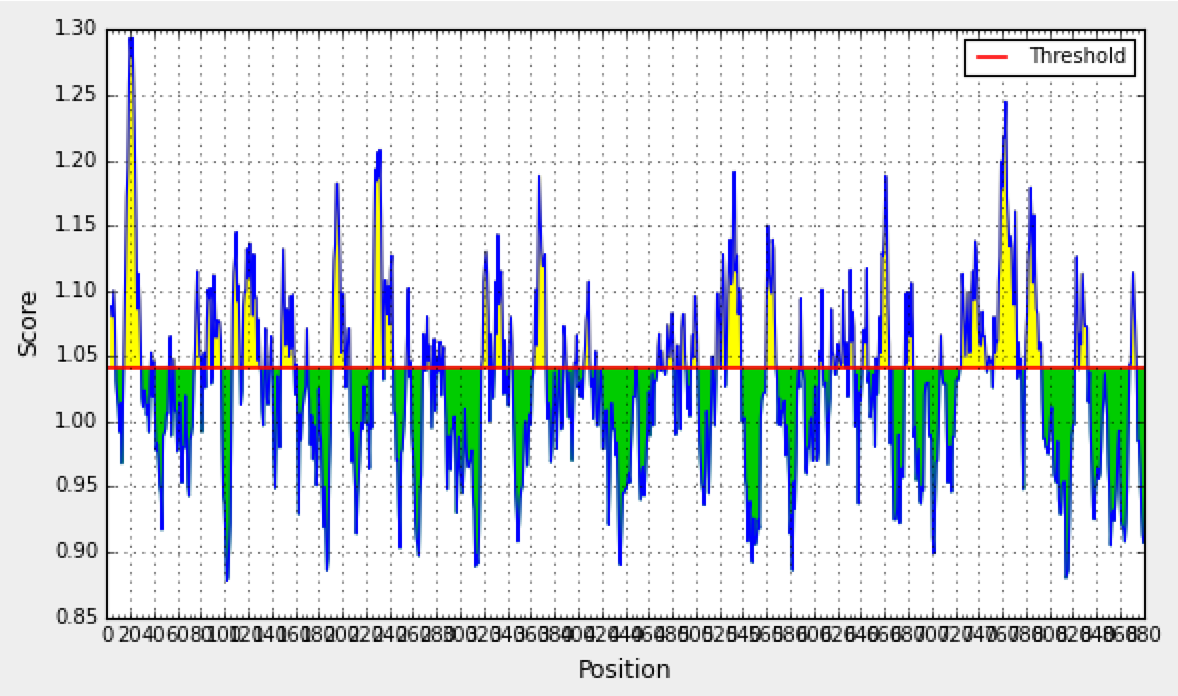

*Regions above redline (threshold) are proposed to be part of B cell epitope

Population coverage calculation

The population coverage must be taken into a different set of alleles to cover all regions and to get desirable immune response in all individuals within a given population. For that reason, all promising MHC-I and MHC-II epitope candidates were assessed for population coverage against different geographic areas through IEDB population coverage calculation tool.

population/area	Class I		
	coverage[a]	average_hit[b]	pc90[c]
China	32.73%	1.08	0.15
Czech Republic	50.80%	2.4	0.2
Germany	58.38%	2.83	0.24
India	41.46%	1.57	0.17
Italy	62.31%	3.07	0.27
Japan	49.38%	2.2	0.4
Korea; South	48.56%	1.8	0.19
Malaysia	22.41%	0.87	0.26
Russia	49.22%	2.23	0.2
South Africa	32.15%	1.28	0.15
Sudan	40.22%	1.73	0.17
Taiwan	39.73%	1.69	0.17
United States	44.84%	1.97	0.18
World	44.76%	2	0.18
**Average**	**44.07**	**1.91**	**0.21**
**Standard deviation**	**10.14**	**0.6**	**0.06**

[a] projected population coverage
[b] average number of epitope hits / HLA combinations recognized by the population
[c] minimum number of epitope hits / HLA combinations recognized by 90% of the population

ILT virus Protein	Position	CTL Epitopes	MHC binding Affinity	Cleavage	TAP	Combined Score	Antigenicity
Glycoprotein D	81	STAAVTYDY	0.7004	0.9654	2.924	3.2646	**0.8537***
	214	PTAALSGQY	0.5548	0.9281	2.494	2.6194	**0.472***
	284	HADDVYRGY	0.5106	0.8916	2.842	2.4439	**0.5214***
	99	LTIPAVGPY	0.3614	0.9319	3.13	1.8308	**0.5037***
	261	TTEQYQTGF	0.3597	0.9576	2.292	1.7856	**0.6102***
	145	FEASVVWFY	0.322	0.918	2.566	1.6333	**0.4547***
	233	QTALVTLEV	0.2998	0.8615	0.477	1.4261	**0.6717***
	79	SQSTAAVTY	0.2602	0.9305	3.203	1.4043	**0.6245***
	188	SAQMWAVDY	0.268	0.5604	2.949	1.3695	0.2221
	281	NTRHADDVY	0.2109	0.5301	2.999	1.1248	**1.1512***

***Antigenic**

ILT virus Protein	Position	CTL Epitopes	Toxicity	Allergenic	Conservancy
Glycoprotein D	81	STAAVTYDY	Non-Toxin	Allergen	100.00%
	214	PTAALSGQY	Non-Toxin	Allergen	100.00%
	284	HADDVYRGY	Non-Toxin	Allergen	100.00%
	99	LTIPAVGPY	Non-Toxin	Allergen	100.00%
	261	TTEQYQTGF	Non-Toxin	Non Allergen	100.00%
	145	FEASVVWFY	Non-Toxin	Non Allergen	100.00%
	233	QTALVTLEV	Non-Toxin	Non Allergen	100.00%
	79	SQSTAAVTY	Non-Toxin	Allergen	100.00%
	188	SAQMWAVDY	Non-Toxin	Allergen	100.00%
	281	NTRHADDVY	Non-Toxin	Non Allergen	100.00%

S.No	Peptide start	Peptide end	CTL epitopes	percentile rank	Allele
1	189	197	AQMWAVDYV	0.12	A*02:01
				0.13	A*02:03
				0.02	A*02:06
				1.1	B*40:01
2	184	192	CAVQSAQMW	0.94	B*44:02
				0.09	B*53:01
				0.27	B*57:01
				0.03	B*58:01
3	410	418	FAAFVACAV	0.7	A*02:03

				0.16	A*02:06
				0.04	A*68:02
				0.23	B*35:01
				0.05	B*51:01
				0.44	B*53:01
4	77	85	FASQSTAAV	0.37	A*02:03
				0.26	A*02:06
				0.1	A*68:02
				1.4	B*07:02
				0.1	B*35:01
5	145	153	FEASVVWFY	0.77	A*26:01
				0.47	A*30:02
				0.89	A*68:01
				1.8	B*40:01
				0.03	B*44:02
				0.04	B*44:03
6	63	71	FLRNAFWTI	0.21	A*02:01
				0.09	A*02:03
				0.11	A*02:06
				1.4	A*23:01
				1.8	A*24:02
				0.3	A*32:01
				0.05	B*08:01
7	269	277	FQGEHLYPI	0.04	A*02:01
				0.06	A*02:03
				0.01	A*02:06
				0.55	B*08:01
8	37	45	FTLFWTCVR	3.9	A*03:01
				2.1	A*11:01
				0.74	A*31:01
				0.11	A*33:01
				0.05	A*68:01
9	413	421	FVACAVALV	0.11	A*02:01
				0.02	A*02:03
				0.02	A*02:06
				0.19	A*26:01
				0.02	A*68:02
				1.2	B*51:01
10	33	41	GAAVFTLFW	1.6	B*44:02
				3.1	B*44:03
				0.26	B*53:01

				0.19	B*57:01
				0.03	B*58:01
11	5	13	HLRRHSRYY	0.22	A*03:01
				0.48	A*26:01
				0.03	A*30:01
				0.49	B*15:01
12	46	54	IMREHICFV	0.13	A*02:01
				0.03	A*02:03
				0.18	A*02:06
				0.08	A*30:01
				0.79	B*08:01
13	99	107	LTIPAVGPY	0.75	A*01:01
				2.2	A*11:01
				0.03	A*26:01
				0.28	A*30:02
				0.03	B*15:01
				0.13	B*35:01
				1.7	B*57:01
				0.46	B*58:01
14	407	415	MPIFAAFVA	1.2	B*07:02
				0.06	B*35:01
				0.18	B*51:01
				0.34	B*53:01
15	401	409	SSNAVSMPI	0.3	A*30:01
				0.04	A*32:01
				0.05	A*68:02
				1.3	B*15:01
				0.29	B*58:01
16	81	89	STAAVTYDY	0.09	A*01:01
				0.18	A*11:01
				0.14	A*26:01
				0.02	A*30:02
				0.24	A*32:01
				0.67	A*68:01
				1.5	B*57:01
				0.59	B*58:01
17	148	156	SVVWFYVIK	1.1	A*03:01
				0.04	A*11:01
				0.35	A*30:01
				1.6	A*31:01
				0.09	A*68:01

18	418	426	VALVGLLVW	1.3	B*51:01
				0.15	B*53:01
				0.11	B*57:01
				0.02	B*58:01
19	405	413	VSMPIFAAF	0.36	A*23:01
				0.2	A*24:02
				1.7	A*30:02
				0.1	A*32:01
				0.04	B*15:01
				0.23	B*35:01
				0.79	B*57:01
				0.11	B*58:01
20	85	93	VTYDYILGR	0.4	A*03:01
				0.05	A*11:01
				0.38	A*31:01
				0.47	A*33:01
				0.11	A*68:01

ILT virus Protein	Position	CTL Epitopes	Toxicity	Allergenic	Conservancy
Glycoprotein D	189	AQMWAVDYV	Non-Toxin	Allergen	100.00%
	184	CAVQSAQMW	Non-Toxin	Allergen	100.00%
	410	FAAFVACAV	Non-Toxin	Non-Allergen	100.00%
	77	FASQSTAAV	Non-Toxin	Allergen	100.00%
	145	FEASVVWFY	Non-Toxin	Non-Allergen	100.00%
	63	FLRNAFWTI	Non-Toxin	Non-Allergen	100.00%
Glycoprotein D	269	FQGEHLYPI	Non-Toxin	Allergen	100.00%
	37	FTLFWTCVR	Non-Toxin	Allergen	100.00%
	413	FVACAVALV	Non-Toxin	Non-Allergen	100.00%
	33	GAAVFTLFW	Non-Toxin	Non-Allergen	100.00%
	5	HLRRHSRYY	Non-Toxin	Non-Allergen	100.00%
	46	IMREHICFV	Non-Toxin	Non-Allergen	100.00%
	99	LTIPAVGPY	Non-Toxin	Allergen	100.00%
	407	MPIFAAFVA	Non-Toxin	Non-Allergen	100.00%
	401	SSNAVSMPI	Non-Toxin	Allergen	100.00%
	81	STAAVTYDY	Non-Toxin	Allergen	100.00%
	148	SVVWFYVIK	Non-Toxin	Non-Allergen	100.00%
	405	VSMPIFAAF	Non-Toxin	Non-Allergen	100.00%
	85	VTYDYILGR	Non-Toxin	Non-Allergen	100.00%

ILT virus Protein	Position	HTL Peptide Sequence	Core peptide	Antigenicity	Toxicity	Conservancy

Glycoprotein D	73	LLSSFASQSTAAVTY	FASQSTAAV	**0.4507**	Non-Toxin	100.00%
	71	IVLLSSFASQSTAAV	LLSSFASQS	**0.4925**	Non-Toxin	100.00%
	219	SGQYLLTLKIGRFAQ	YLLTLKIGR	0.392	Non-Toxin	100.00%
	50	HICFVRNAMDRHLFL	ICFVRNAMD	-0.0321	Non-Toxin	100.00%
	82	TAAVTYDYILGRRAL	VTYDYILGR	**0.6939**	Non-Toxin	100.00%

Interferon (IFN-γ) epitope prediction

The interferon gamma inducing HTL epitopes were predicted by using IFNepitope server. All HTL epitopes were used as an input to identify the presence of the IFN gamma epitope.

Serial No.	Epitope Name	Sequence	Method	Result	Score
1	Epitope_4	HICFVRNAMDRHLFL	MERCI	POSITIVE	1
2	Epitope_1	LLSSFASQSTAAVTY	MERCI	NEGATIVE	1
3	Epitope_2	IVLLSSFASQSTAAV	MERCI	NEGATIVE	1
4	Epitope_3	SGQYLLTLKIGRFAQ	SVM	NEGATIVE	-0.16843448
5	Epitope_5	TAAVTYDYILGRRAL	SVM	POSITIVE	0.58974753

B cell epitopes identification

Linear B cell epitopes

The linear/continuous B cell epitopes were identified using IEDB. Further the predicted B cell epitopes were also assessed for surface accessibility and antigenicity.

Prediction of B cell epitopes by Emni surface accessibility

S.No.	Start	End	Predicted Peptide	Length	Emini Score
1	4	14	PHLRRHSRYYA	11	2.24
2	106	111	PYNRYL	6	1.885
3	136	141	AKEKEK	6	4.634
4	262	267	TEQYQT	6	3.241
5	280	285	TNTRHA	6	1.724
6	287	292	DVYRGY	6	1.128
7	302	318	LLRKKNPSAPDPRPDSV	17	
8	327	352	KKAEGRTPDAESSEKKAPPEDSEDDM	26	
9	365	388	PEDDEVPEDTEHDDPNSDPDYYND	24	

10	396	401	EETTKS	6	3.201

Prediction of B cell epitopes by Kolaskar and Tongaonkar antigenicity prediction

No.	Start	End	Predicted Peptide	Length	Antigenicity Score
1	28	46	KRCCSGAAVFTLFWTCVRI	19	
2	49	55	EHICFVR	7	
3	70	77	TIVLLSSF	8	
4	82	90	TAAVTYDYI	9	
5	98	105	ALTIPAVG	8	
6	115	124	SRGCDVVELN	10	
7	146	155	EASVVWFYVI	10	
8	177	188	DVQLLSECAVQS	12	
9	194	201	VDYVPSTL	8	
10	219	226	SGQYLLTL	8	
11	234	240	TALVTLE	7	
12	253	258	NFLPSK	6	
13	272	277	EHLYPI	6	
14	319	326	PQEIPAVT	8	

Prediction of B cell epitopes by Emni surface accessibility

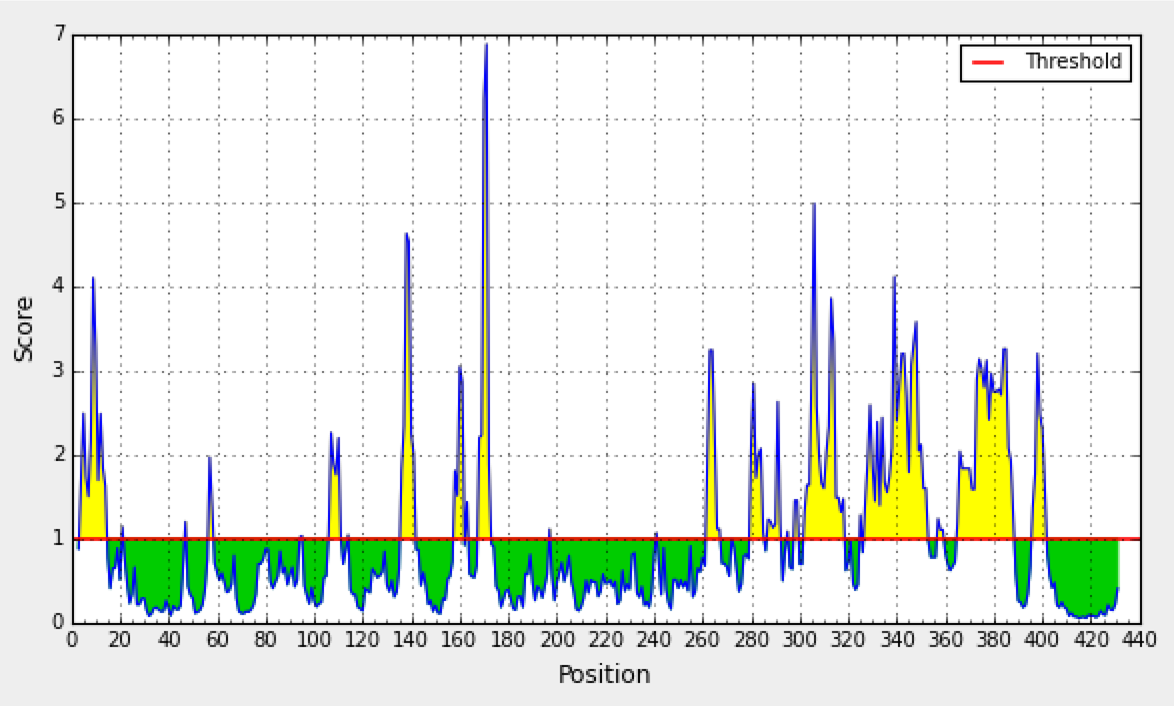

*Regions above redline (threshold) are proposed to be part of B cell epitope

Prediction of B cell epitopes by Kolaskar and Tongaonkar antigenicity prediction

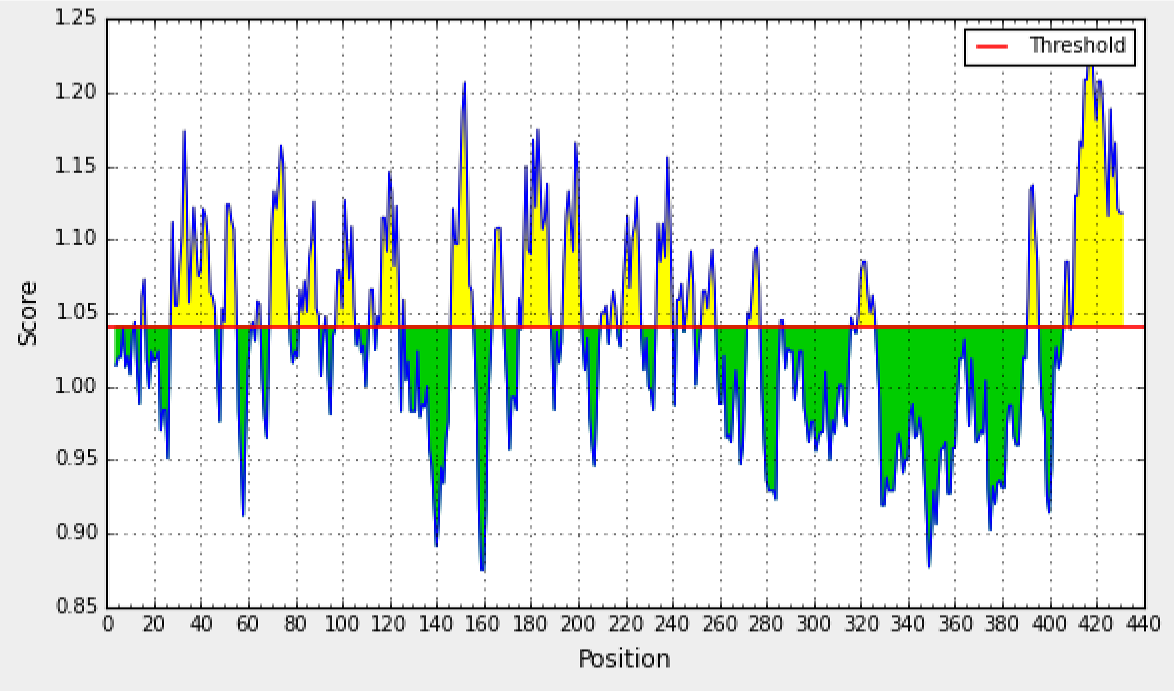

*Regions above redline (threshold) are proposed to be part of B cell epitope

Final vaccine construct of predicted CTL and HTL epitopes of Glycoprotein B and D of ILT virus

GIINTLQKYYCRVRGGRCAVLSCLPKEEQIGKCSTRGRKCCRRKKEAAAKYVFNVTLYYAAYYVTRVPID

|-------------------------------- β defensin – Adjuvant--------------------------|-Linker|------B CTL---|Linker|---B CTL

YAAYTSVDCVVEYAAYDTVEISPFYAAYATEAIDGIFAAYMSHGLAEMYAAYSTENHNLDIAAYLLGENY

-|Linker|---B CTL---|-Linker|---B CTL-|Linker|--B CTL---|Linker|---B CTL------Linker---B CTL---Linker---B CTL

LLYAAYLRDTGTLNYAAYSTAAVTYDYAAYPTAALSGQYAAYHADDVYRGYGPGPGGFLIAFQKLMSHG

------Linker---B CTL---Linker---D CTL----Linker---D CTL------Linker---D CTL--------Linker--------B HTL---------

LAGPGPGHRRYFLLGENYLLYEGPGPGVSGIASFLSNPFAALGPGPGFKYVMNLKSNPVQVLGPGPGHICF

----Linker----------B HTL-------------Linker---------B HTL-------------Linker--------B HTL--------------- Linker---------

VRNAMDRHLFLGPGPGTAAVTYDYILGRRALHHHHHH

---D HTL---------- Linker--------D HTL------------- HIS TAQ

CodonUsage adapted to Escherichia coli (strain K12)

Improved DNA:

```
GGTATCATCAACACCCTGCAGAAATACTACTGCCGTGTTCGTGGTGGTCG    50
TTGCGCTGTTCTGTCTTGCCTGCCGAAAGAAGAACAGATCGGTAAATGCT    100
CTACCCGTGGTCGTAAATGCTGCCGTCGTAAAAAAGAAGCTGCTGCTAAA    150
TACGTTTTCAACGTTACCCTGTACTACGCTGCTTACTACGTTACCCGTGT    200
TCCGATCGACTACGCTGCTTACACCTCTGTTGACTGCGTTGTTGAATACG    250
CTGCTTACGACACCGTTGAAATCTCTCCGTTCTACGCTGCTTACGCTACC    300
GAAGCTATCGACGGTATCTTCGCTGCTTACATGTCTCACGGTCTGGCTGA    350
AATGTACGCTGCTTACTCTACCGAAAACCACAACCTGGACATCGCTGCTT    400
ACCTGCTGGGTGAAAACTACCTGCTGTACGCTGCTTACCTGCGTGACACC    450
GGTACCCTGAACTACGCTGCTTACTCTACCGCTGCTGTTACCTACGACTA    500
CGCTGCTTACCCGACCGCTGCTCTGTCTGGTCAGTACGCTGCTTACCACG    550
CTGACGACGTTTACCGTGGTTACGGTCCGGGTCCGGGTGGTTTCCTGATC    600
GCTTTCCAGAAACTGATGTCTCACGGTCTGGCTGGTCCGGGTCCGGGTCA    650
CCGTCGTTACTTCCTGCTGGGTGAAAACTACCTGCTGTACGAAGGTCCGG    700
GTCCGGGTGTTTCTGGTATCGCTTCTTTCCTGTCTAACCCGTTCGCTGCT    750
CTGGGTCCGGGTCCGGGTTTCAAATACGTTATGAACCTGAAATCTAACCC    800
GGTTCAGGTTCTGGGTCCGGGTCCGGGTCACATCTGCTTCGTTCGTAACG    850
CTATGGACCGTCACCTGTTCCTGGGTCCGGGTCCGGGTACCGCTGCTGTT    900
ACCTACGACTACATCCTGGGTCGTCGTGCTCTGCACCACCACCACCACCA    950
C
```

CAI-Value of the improved sequence: 1.0

GC-Content of the improved sequence: 54.25867507886435
